# A comprehensive walkability evaluation system for promoting environmental benefits

**DOI:** 10.1038/s41598-023-43261-0

**Published:** 2023-09-27

**Authors:** Ilho Jeong, Minje Choi, Juhyeon Kwak, Donggyun Ku, Seungjae Lee

**Affiliations:** 1 Department of Transportation Engineering/Department of Smart Cities, University of Seoul, Seoul, South Korea; 2 University of Cambridge, Cambridge, United Kingdom; 3 Department of Transportation Engineering, University of Seoul, Seoul, South Korea

**Keywords:** Environmental impact, Climate-change adaptation, Climate-change impacts, Climate-change policy, Environmental impact, Civil engineering, Climate change

## Abstract

Pedestrian-oriented urban strategies such as the Paris 15-minute City are needed to respond to the global boiling. Quantitative evaluation of pedestrian-oriented urban objectives is important for various cities, and in this paper, a walkability evaluation system for the advanced model is developed considering the characteristics of a large city. The system calculates the walkability of Seoul. The evaluation system uses the Betweenness index as a weight in the urban network analysis. Considering stations with a high betweenness in urban traffic is essential for evaluating a pedestrian-oriented metropolis. Our findings in this study are that the UNA index in WES is critical for transit-oriented, walkable cities. The large city needs to find the location for mobility hubs or stations to observe the last mile. Installing a mobility hub or station at a high-value location in the city center is functionally important. In a pedestrian-oriented city, citizens can walk and bike the last mile in a busy city center. Walkable cities can encourage active transport and ultimately create more sustainable and environmentally friendly transportation systems. This study offers valuable insights into pedestrian infrastructure, urban systems, and policies that promote green transportation.

## Introduction

Urban models for walkable cities and walkability are being presented all over the world. The Paris 15-minute City is an example of a walkable city strategy that responds to global boiling. The 15-minute City^1–5^ is an urban planning concept that was first presented by Carlow Moreno in 2016^5^. The key elements of this model have been described as “chrono-urbanism” by Carlos Moreno. The 15-minute City is an urban planning concept in which most daily necessities and services, such as work, shopping, education, healthcare, and leisure, can be easily reached by foot or bike from anywhere in the city within 15 min^6^. The model prioritizes improved temporal and spatial proximity and accessibility. The model enables individuals to conveniently access essential urban amenities and opportunities within a 15-min walking distance^7^. The walkable city model is a vital strategy for citizens’ lives. Walking is inherently linked with public transportation. A related concept, the superblock or car-free space, was introduced in Barcelona in 2016^8^. Transformation in Barcelona improved pedestrian mobility due to the reduction of traffic found in these urban areas^9^. Portland’s 20-min city concept evolved from the 20-min Neighborhood concept. Ideally, a city concept that can be reached on foot, by bicycle, public transport^10^.

Large cities consist of multiple city centers, and public transportation plays a crucial role. The 15-minute City model is a polycentric settlement system able to offer essential and primary services in a few minutes^11^. As a representative metropolis, Seoul’s development has resulted in enhanced public transportation access and the emergence of secondary urban centers, effectively reducing commuting times. Enhanced public transportation leads to shorter commutes in high-density urban areas^12^. Seoul is six times larger than Paris. Implementing walking and cycling-focused policies like 15-minute City is not immediately feasible in Seoul. Public transportation must also be considered, as it serves as a mediator for city-wide accessibility. Therefore, large cities such as Seoul should construct a public-transport-connected, pedestrian-focused urban model. Furthermore, cities with transit connections and walkable infrastructure expand the scope of activities available to their citizens. Cities that prioritize pedestrian infrastructure and are well-connected by transit can transform themselves into walkable metropolises.

Seoul has implemented a 30-min walkable city plan that enables individuals to access various urban facilities, including public transportation, mobility hubs, offices, and parks within walking distance. The city is working towards decarbonization by proposing a walking-centered urban restructuring policy. This paper proposes an evaluation system for a walking-centered city, which considers factors such as sidewalks, urban furniture, buildings, and more.

Global warming is changing to global boiling. As the climate crisis intensifies, the creation of cities resilient to climate change is a top priority. Many countries, including Korea, have announced policies to achieve net-zero emissions by 2050. Transport activities are major contributors to global greenhouse gas (GHG) emissions. Transportation emissions will increase at a faster rate than other energy end-use sectors, partly due to economic growth and an increase in population^13^. With the rise in environmental awareness, both policymakers and researchers are increasingly interested in sustainable mobility planning approaches, including the promotion of non-motorized and eco-friendly mobility^14^. The UN has set SDGs (Sustainable Development Goals, Global Goals) as a common goal of the international community, and research and policies on the walkability index can help not only the “Climate Action” of SDGs but also overall common goals.

Policies for decarbonization are being presented around the world, and a representative policy for transportation is a city centered on activating public transportation, improving accessibility, and walking. Energy efficiency improvement and CO_2_ reduction are important issues about automotive and transportation^15^. To make a more sustainable society, cleaner transport technologies such as zero-emission vehicles and sustainable mobility are being investigated, promoted and supported by various policy instruments^16^. When environmental pollution becomes serious problem and contributes significantly to global warming, zero-emission transport systems become necessary^17^.

Walkability is a measure of the extent to which the walking environment is suitable for walking and can predict levels of human physical activity and active mobility^18,19^. Walkability is defined as the degree to which a walking environment is familiar to people who walk to work^19,20^. Walkability is a measure of whether the built environment of a neighborhood encourages pedestrian activity. There are two main reasons why walkability is becoming increasingly valued. The first reason is that walkable cities promote balanced urban development and public services while providing people with a better place to live, which leads to improved neighborhood satisfaction^21^. Second, walking benefits people’s physical and mental health^22,23^.

There are several ways to measure walkability. Walkability, which is a combination of net residential density, intersection density, commercial floor area ratio, and land use composition, is the most widely used measurement method^24^.

Many studies have attempted to address the various essential factors that contribute to walkability. Residential density, street connectivity, traffic conditions, and aesthetics affect walking comfort^25^. Walkable distance, dwelling density, and high land-use mix were positively related to walking^26^. Population density and pedestrian infrastructure were related to an increased propensity to walk^27^. Studies investigating the association between neighborhood environment characteristics and attributes and walking also considered objective as well as subjective measures^28^. Marshall et al. studied the relationship between walkability and air pollution and concluded that neighborhoods with high walkability tended to have higher levels of primary traffic-related pollution (NOx) but lower ozone concentrations^29^. The issues related to walkability, including factors that affect walkability and ways to improve it, not only have a direct impact on the satisfaction of citizens’ commuting and leisure travel but are also connected to city policies, environment, and health, making it more practical to apply them. Careful consideration is needed to ensure that in this study, the methodology applied in the existing literature is advanced to calculate walkability considering factors that more directly affect walking, such as walking network and gradient.

**Figure 1:**
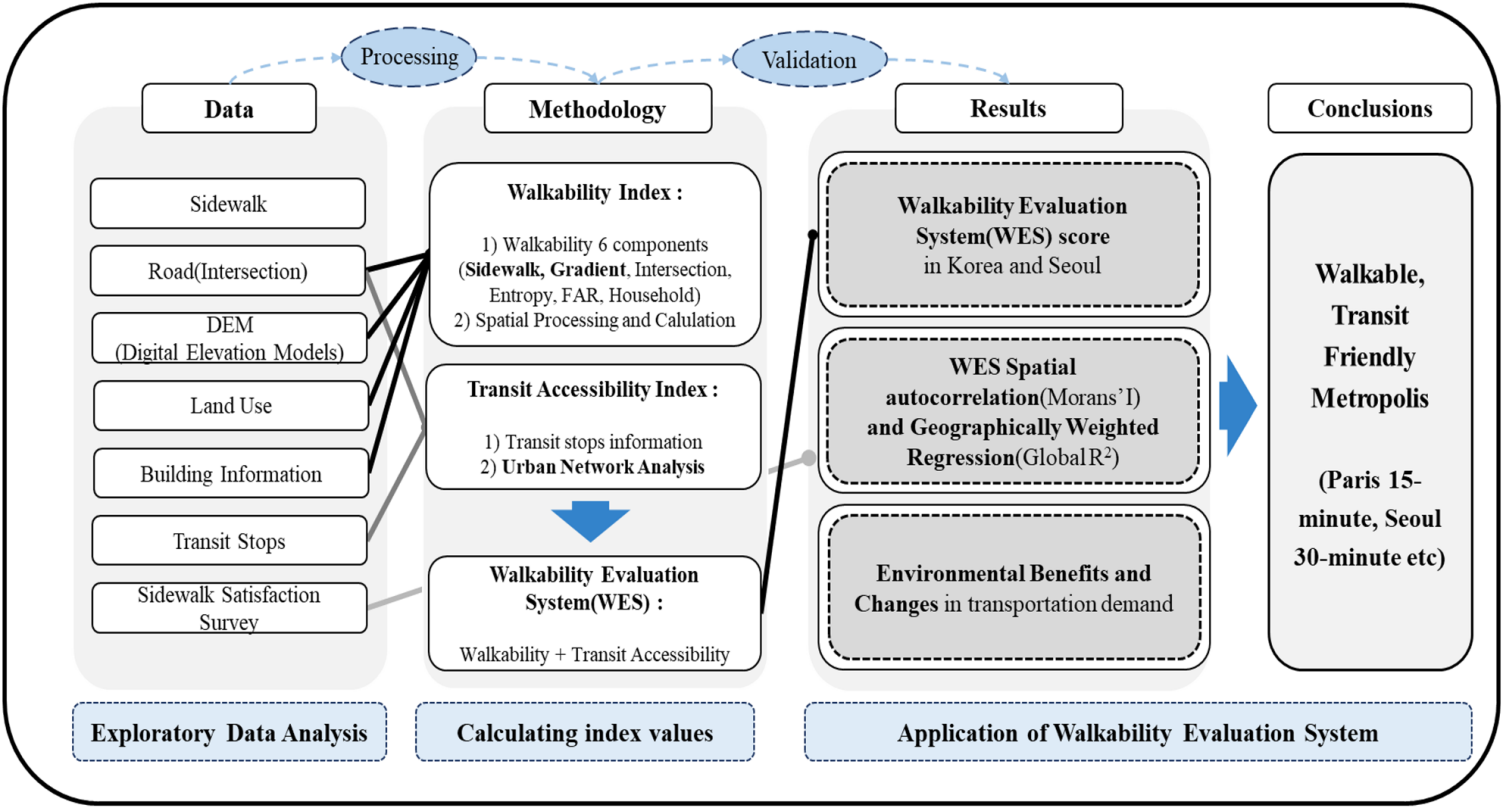
Framework of this study.

This study aims to develop an evaluation system by calculating pedestrian evaluation indicators based on various urban big data for the implementation of pedestrian-centered cities such as the 15-minute City example. It aims to develop a system that can quantitatively evaluate pedestrian-centered policies. An evaluation system is developed that reflects the representative case of a pedestrian-centered city and the characteristics of Seoul, which is centered on public transportation. It aims to analyze traffic and environmental impact through the evaluation system. Pedestrian networks and information, intersections, elevations and slopes, land use, building information, public transit stops, and pedestrian satisfaction data were used in this study. Unlike other papers that mainly reflect architectural environmental factors that affect walkability, this paper directly reflects pedestrian environments such as walking paths and slopes. The characteristics of public transportation as access traffic were considered. A comprehensive pedestrian evaluation system was established considering the network centrality of public transportation. In this study, environmental and health benefits associated with the final evaluation system were derived. The practical benefits of improving walkability were calculated and propose policies for each element in Fig. 1.

## Literature review

To create a walkability evaluation system that can realistically reflect walking and public transportation, appropriate collection of urban data, walking data and public transportation data is required. The collected data requires a process of calculating the walkability index (WAI) and transit accessibility (TA). TA should also consider network centrality. The role of public transport in mediating traffic was considered by considering the betweenness index. The literature review was organized in the order of Walkability and Walkability Index, Urban Network Analysis and Centrality, and Environmental Benefits. The order of review is the same as the order of analysis in this study. First, the walkability index is calculated. Public transport accessibility is calculated through city network analysis. Add WAI and TA to create a walkability evaluation system. The environmental benefits are then calculated.

### Walkability and walkability index

In general, the walkability index is constructed by considering transportation infrastructure such as land, dwelling, and intersection density. The walkability index developed by Frank has been used at various geographical scales; census divisions, and network buffers around specific households or commercial centers^30,31^. Frank et al. constructed a walkability index by selecting the most explanatory model for significant figures of daily activity time (minutes). The model consists of the sum of land use, residential density, and intersection density, and A weight was tried for lane use variable^32^. When evaluating walkability, an index classification method such as the 6D framework is sometimes applied. 6D consists of Density, Diversity, Design, Destination accessibility, Distance to transit, and Demand management^33^. Generally, the well-known walkability index is a summation value of z-scores of land use, residential density, FAR (floor area ratio), and intersection density. The walkability index consisted of the connectivity index (intersection density), entropy index (land use degree), FAR (floor area ratio, commercial facilities), and household density. All partial indices are calculated separately for each city district to calculate the walkability index^34^. The walkability index was calculated based on Frank et al.’s formula not including the z-score for the retail floor area, and z-scores for walkability were then classified with quantiles (five classes of equal intervals divided into very high, high, moderate, low and very low walkability neighborhood groups)^35^. The walkability index was calculated based on Frank et al.’s formula not including the z-score for the retail floor area, and z-scores for walkability were then classified using quantiles (five classes of equal intervals divided into very high, high, moderate, low and very low walkability neighborhood groups)^36^. The walkability index was determined from measures of population density, street connectivity, and food and physical activity resources measured from participants’ pre and post-move residential locations^37^. Frank et al. calculated the sum of z-scores of land-use mix and net residential and intersection density^18^. Frank et al. calculated the sum of z-score land-use mix, net residential density and intersection density and divided them into quartiles (lowest quartile, second quartile, third quartile and highest quartile)^38^. Possible destinations are weighted based on, distance, size and importance. The importance and desirability of a set of possible destinations are derived from Banerjee et al. (1984) who ranked residents’ views of given destinations; e.g., ‘everyday’ destinations such as post offices, pharmacies and food stores rank higher than sports arenas or night clubs. The sum of the weighted intersection z-score and ‘everyday’ retail z-score combine to form the walk opportunities index. As the walk opportunities index considers different types of individual businesses, as well as intersection types, it likely explains more walking behavior than the WI^39^.

The walkability index (WAI) was established by constructing an index considering the characteristics of walking networks and slopes directly related to walking. By utilizing big data from various cities, walking networks and elevation data are integrated to identify more ‘walkable’ areas, thereby upgrading the previously studied walkability index. Compared to Frank’s model, it is an index that includes more walking information and public transportation information. Among the 6D frameworks, Density, Diversity, Design, and Destination accessibility were reflected. Distance transit is reflected as the number of weighted stops.

### Urban network analysis and network centrality

Urban network analysis and network centrality numerically analyze the characteristics of structural networks that consist of nodes and links. Network centrality measurements are conducted using social network analysis, which can be applied to man-made networks such as railway and road networks. It provides insight into the importance of railway stations and road intersections^40^. Network analysis to identify critical nodes is a fundamental problem. A study on network node centralization can provide a theoretical basis for public transportation system planning^41^. Relative node importance evaluation includes various metrics such as Degree, Betweenness, Closeness, and Straightness. Degree centrality describes the possible movement activity that a mover can obtain from a station, and moderate centrality describes the ability to control a station’s movement activity. Degree centrality indicates the independence or efficiency of a station. Centrality characteristic analysis is useful for urban railway management and operation, and is the basis for analyzing network vulnerabilities that are important for the safety of urban railway systems^42^. Degree centrality provides the concept of the number of connections possessed by a vector^43^. The most common measure of node importance is betweenness centrality, which is the proportion of nodes acting as bridges for important transfers between pairs of nodes along the shortest path within a network^44^.

Betweenness centrality is particularly relevant for public transportation. Stations can be used extensively because they are near important locations, however, other stations can be used much more because they serve as transfer points to multiple locations^45^. Betweenness centrality is a widely used centrality index in UNA that can be used to describe the importance of that node in terms of the shortest path. We measured the importance of nodes in terms of connecting them to other nodes via the shortest path^46^. Betweenness centrality is a measure of the importance of a node within a network. Degree centrality is based on the idea that important nodes have the greatest number of connections in a graph. Closure centrality measures the proximity of a node to all other nodes along the shortest path. The preferred path between two points on a network is defined by minimizing the total impedance of every link^47^. Within the public transport network itself, the Betweenness centrality index will play a major role as a bridge, and in this study, it will also play an important role in pedestrian-public transport travel. Transit considerations are necessary for the advanced 15-min model where walking and transit are central. Stations with a high transit-betweenness centrality play a transit role in the network. Improving transit or installing transit facilities in areas with high betweenness can contribute to the revitalization of public transportation. In this study, transit stops and the number of Betweenness centrality weighted transit stops in the region are calculated and used as an integrated index called Transit Accessibility. The betweenness index is considered as the main measure of the link between walking and public transport. Installing mobility hubs^48^ or transit facilities in areas with high index value in Seoul will contribute to the achievement of n-minute cities in large cities.

### Environmental benefits

Many methods are used to evaluate emission reduction by active transport. Many people underestimate actual reductions by assuming that a mile of walking or bicycling reduces just one vehicle-mile, ignoring leverage effects^49^. Installing sidewalks on all streets in a typical North American community reduces about 12 motor vehicle miles per additional mile walked or biked, and active modes tend to substitute for short trips that have high emission rates due to cold starts and congestion^50^.

The Global High Shift Cycling Scenario estimates that dramatically increasing bicycle and e-bike use to serve all consumer demands could reduce up to 11% of urban transportation emissions. Active transportation improvements that result in residents achieving physical activity targets (150 weekly minutes for physical activity) could reduce transportation emissions 24% and avoid 167,000 deaths and gain 2.5 million disability adjusted life years, with $1.6 trillion monetized health benefits^51^. An analysis of travel activity in seven European cities found that increased walking and bicycling significantly reduce motorized travel and per capita carbon emissions^54,55^. An average person who shifts from driving to bicycling one daily trip 200 days a year decreases approximately 0.5 tons of annual CO2 emissions, a substantial reduction of per capita GHG emissions.

Ngo used before-and-after travel surveys conducted from 2012 to 2015 to measure the vehicle travel, emissions, health impacts of the Comox–Helmcken Greenway, a 2-km pedestrian and bicycle pathway in downtown Vancouver, British Columbia. The results indicate statistically significant reductions of − 22.9% for average daily motorized GHG emissions, − 23.7% for energy consumption^56^.

A fuel life cycle scenario analysis was conducted to evaluate the environmental benefits of a “road-to-rail” policy, using 2017 as the base year. All “road-to-rail” scenarios achieved energy savings and emission reductions compared to freight transport^57^. Published a study on the impact of traffic emissions on air quality in Lisbon, treating major roads as route sources. Road emissions were calculated based on average daily traffic volume, and emission factors were classified according to road class and vehicle type^58^. The environmental benefit of the new PM-only (personal mobility-only) road was calculated using the average conversion method and the traffic quota method. The benefit was calculated as a reduction in pollutant emissions due to a decrease in car traffic^59^. Considering the global effort for new vehicle technologies, a review of the expected environmental and traffic noise impacts when these types of vehicles are introduced to the market has been provided^60^.

Integrating active transportation contributes to health-enhancing physical activity for commuters from a public health perspective^61^. In this study, the purpose of this study was to induce the use of public transportation by improving public transportation accessibility and to improve the health of users through the vitalization of walking. Reducing vehicle movement by approximately 223 million km/year, a reduction of 116 deaths from increased physical activity and 5.6 deaths from air pollution can be achieved. In terms of the economy, the health effect is expected to reach net savings of approximately $ 200 million per/year^62^. In this study, health benefits can be calculated based on the results of analyzing the effect of switching to public transportation due to public transportation infrastructure improvement. It could improve the health of the public when car use decreased and bicycle and public transportation use increased; thus, the benefits of increasing commuters’ physical activity level, reducing air pollution, and reducing the risk of traffic accidents were calculated^63^.

### Research gaps and contributions

This study advances the walkability index for evaluating advanced walkable cities, such as the 15-minute City by utilizing various urban big data. It considers the geometrical characteristics of pedestrian paths in a specific area as well as the density of intersections, which are considered indicators of the connectivity of areas. The UNA betweenness index considers the intermediary nature of public transport. The system was constructed considering the indicators that mediate transport. The UNA indicator identifies the last mile and the 15-min urban area. The slope of each area of the city that could affect active travel and walking was calculated and included in the Walkability Index (WAI). The walkability evaluation system (WES), which integrates WAI and TA, is built by calculating Transit Accessibility (TA) considering the network centrality of bus, subway, and bicycle stops. The constructed WES is verified through geo-weighted regression analysis and local coefficients can be confirmed. Environmental benefits are calculated by combining WES improvements with public transport changes. Also, as one of the case studies, the effect of Seoul’s Safety Speed 5030 policy is analyzed. WES provides issues worth reviewing in analyzing various aspects of walking policy and urban planning.

## Methods

**Figure 2:**
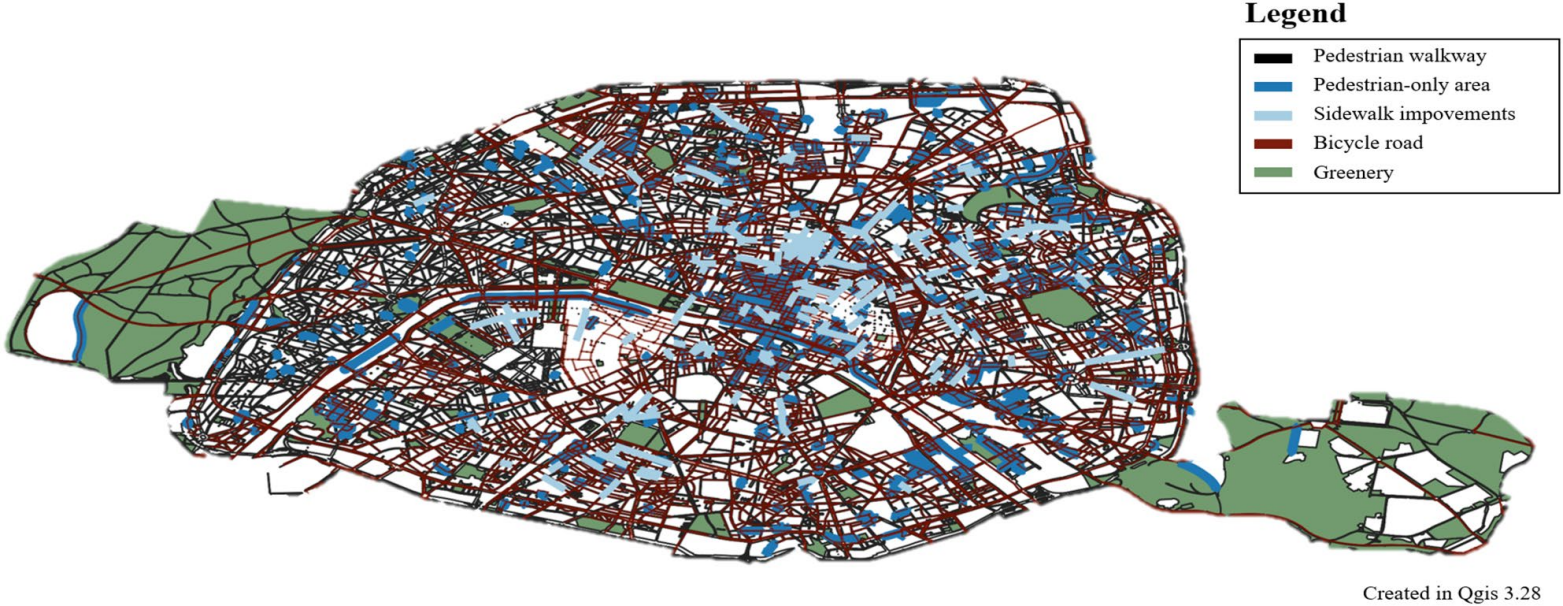
Paris, 15-minute City, this map is based on data from Paris Data (https://opendata.paris.fr/), this map was generated in Qgis desktop version 3.28 software (https://qgis.org/ko/site/).

**Figure 3:**
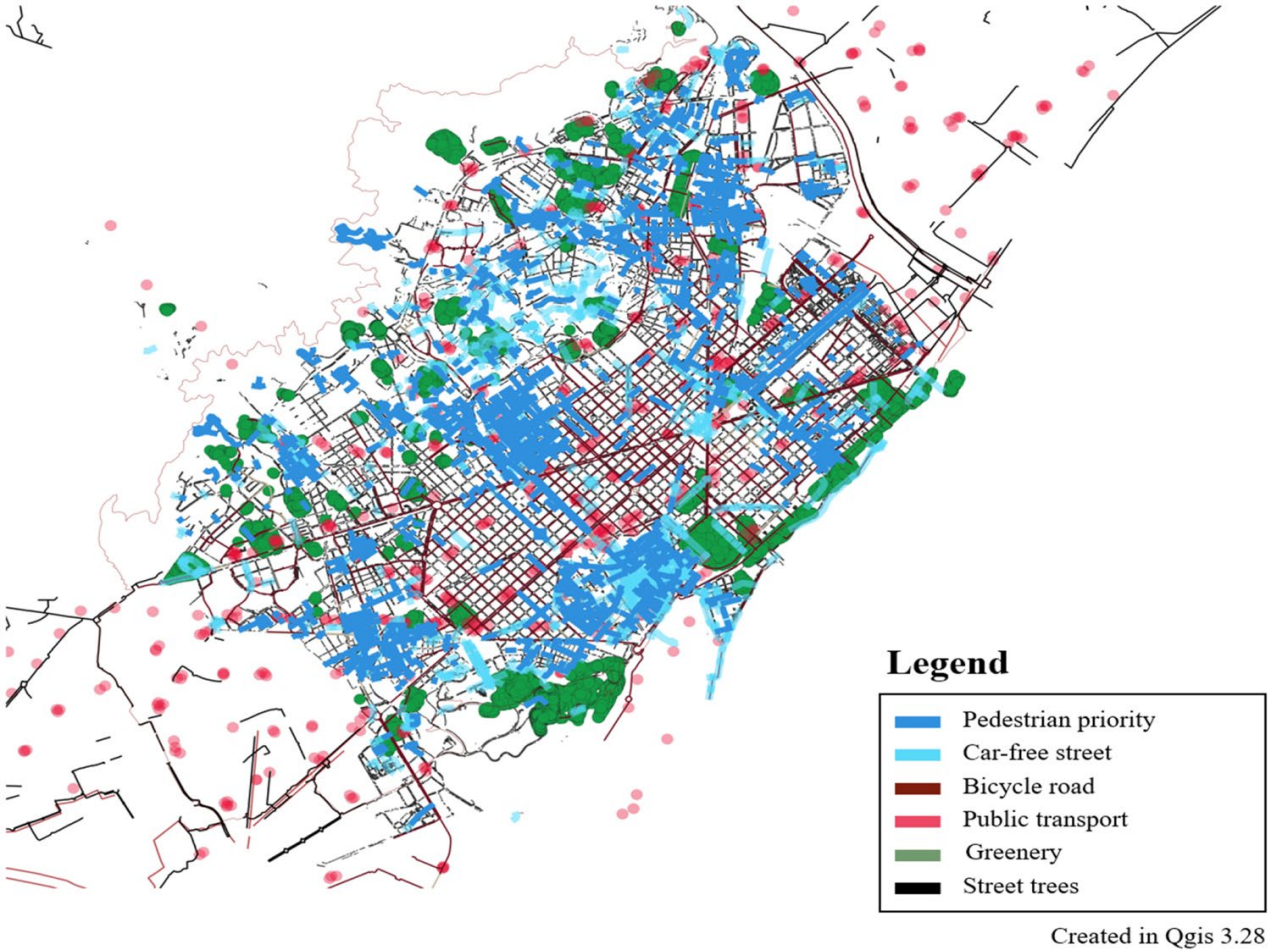
Barcelona, Superblocks, this map is based on data from Open Data BCN (https://opendata-ajuntament.barcelona.cat/en/), This map was generated in Qgis desktop version 3.28 software(https://qgis.org/ko/site/).

**Figure 4:**
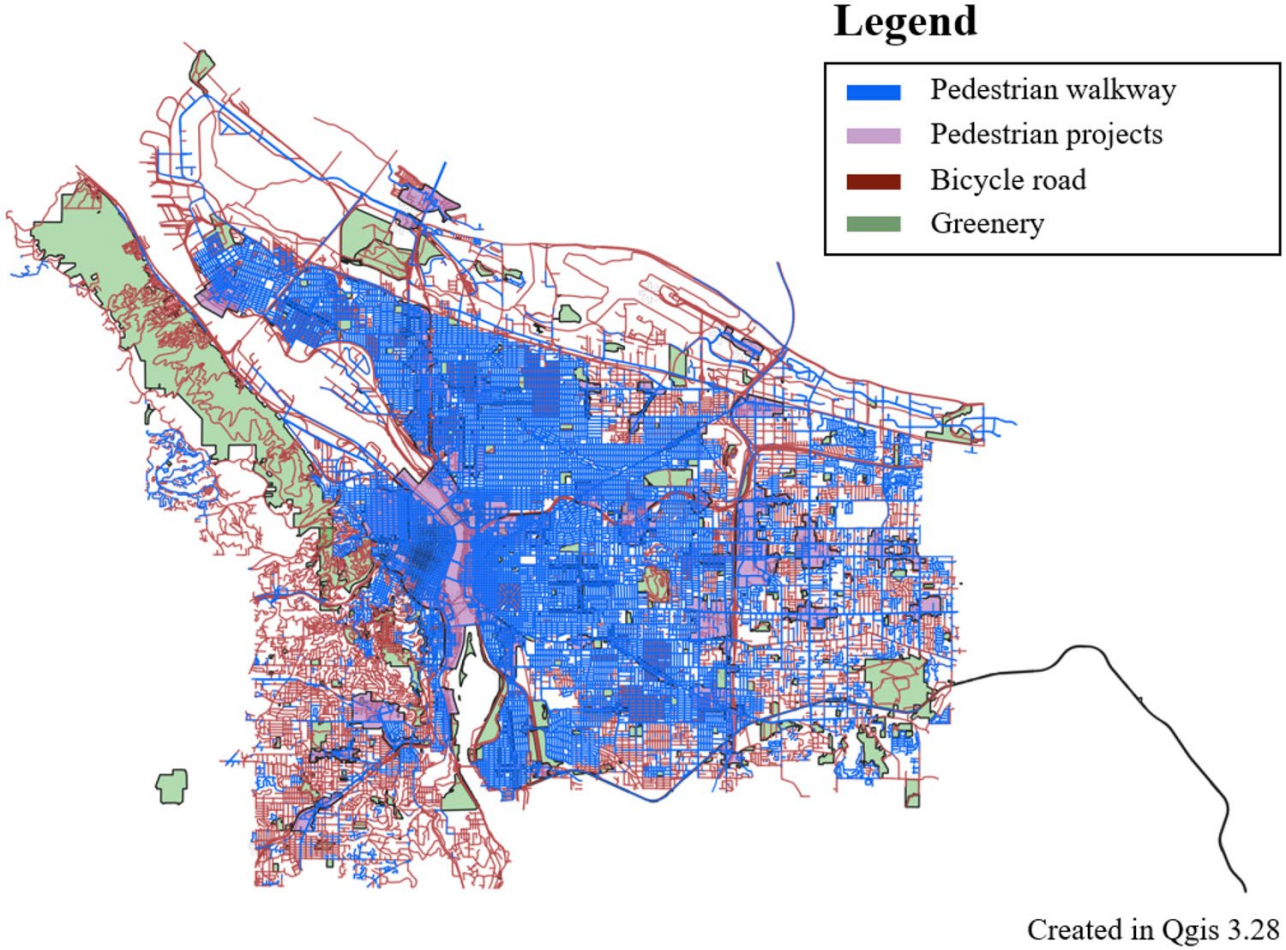
Portland, 20-min city, this map is based on data from PortlandMaps-Open Data (https://gis-pdx.opendata.arcgis.com/), This map was generated in Qgis desktop version 3.28 software (https://qgis.org/ko/site/).

The pedestrian networks of Paris, Barcelona, and Portland are as follows (Figs. 2, 3, 4). Pedestrian networks in each city are developed following the pedestrian policy (Table 1). Paris aims to provide access to a wide range of service facilities within 15 min, with a focus on walking and cycling^5,64^. Barcelona permits automobile traffic only on the outer main roads and pedestrians on the inner roads^9,65^. Portland aims to provide easy access to services and transit facilities by walking and biking^66,67^.

**Table 1:** Pedestrian policy cities examples.

City	Model	Contents	Transportation	Measures
Paris	15 minute City	Seven dimensions constitute the 15-minute City: (1) proximity, (2) density, (3) diversity, (4) Digitalization, (5) human-scale urban design, (6) flexibility, (7) connectivity^64^	Walking, Bike^64^	Proximity of service, social interaction, health benefits, environmental benefits^5^
Barcelona	Superblocks	9 blocks (400 m × 400 m), street rest area, convenience facilities, exercise space, wellness^65^	Restrictions on arterial roads for vehicles, Pedestrians on internal roads^65^	Environmental benefits from traffic, Pedestrian mobility^9^
Portland	20 minute City	Businesses, frequent transit service, schools, parks or greenspaces and other amenities close enough to safely and easily walk or bike^66^	Walking, bike^67^	% of the population within 1/2 mile of a grocery store, a park, an elementary school, and frequent transit^66^

**Figure 5:**
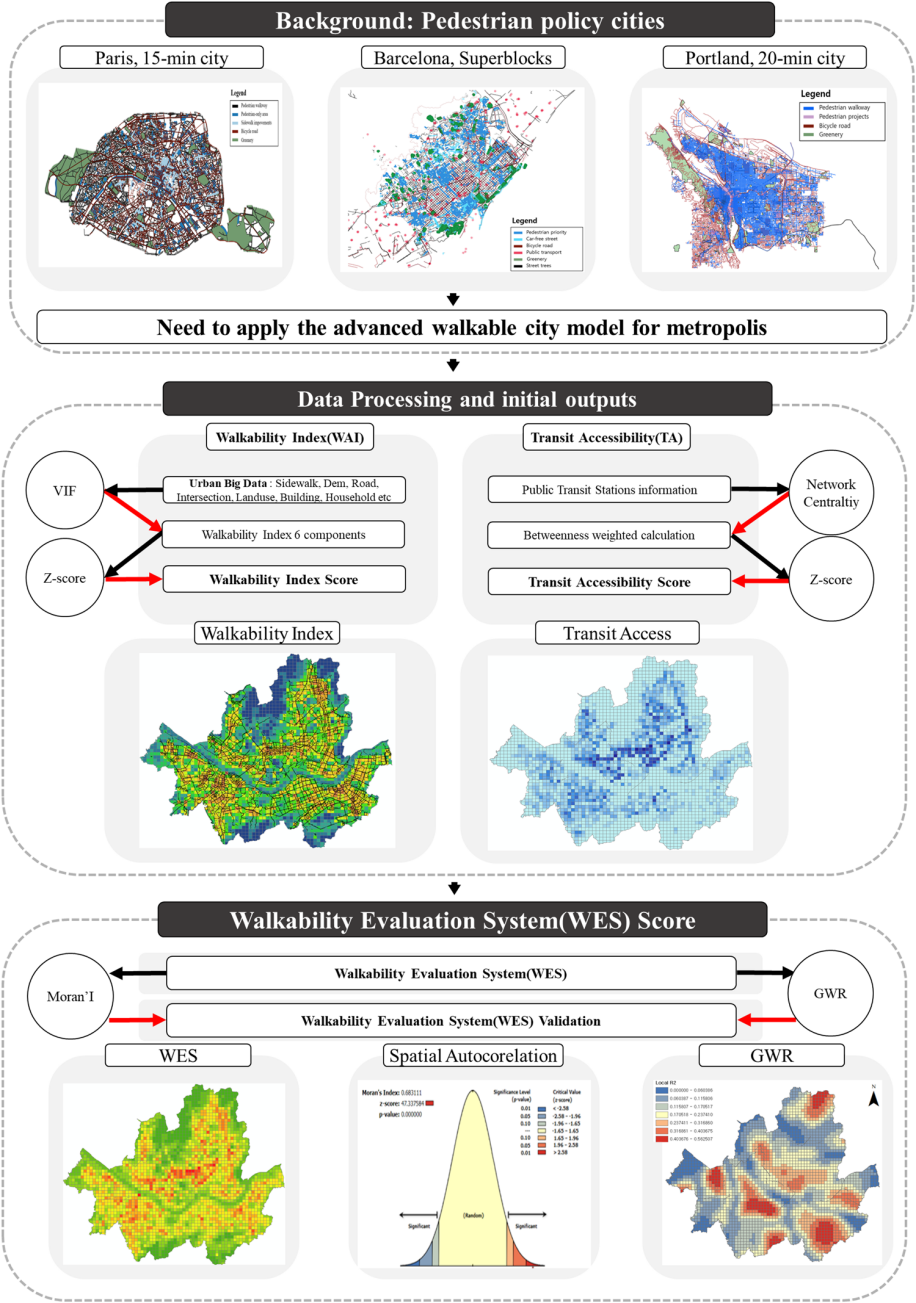
Methodology of walkability evaluation system (WES).

The limitation is that the Paris 15 minute transport policy may be difficult to apply to other cities. Barcelona considered the effect (environmental) of vehicle traffic restrictions in the city center. Portland considered access to public transit facilities. Seoul needs to apply a different model from cities with developed tourism and shopping. Seoul needs to apply the x-min city concept expanded from Paris, Barcelona and Portland. Therefore, Seoul should, first of all, have a walking and cycling-oriented orientation. Second, it should include policies restricting vehicle traffic in the city center. Third, the connection between public transportation and walking should be considered. In this paper, we construct WES considering walking, cycling and public transportation. In addition, the relationship between the safety speed 5030 and walkability, which restricts vehicle traffic in the city, is partially explained. In this study, a walkability evaluation system (WES) is developed by utilizing various big data of the city. The index considers the geometrical characteristics of pedestrian paths, the density of intersections, and the slope of each area to calculate the walkability index (WAI). The study also calculates Transit Accessibility (TA) by considering the network centrality of bus, subway, and bicycle stops. The WES is built by integrating WAI and TA and is verified through geo-weighted regression analysis. The study combines WES and public transport to estimate environmental and health benefits related to increased walkability. Further analysis can be performed to directly assess the impact of walkability, and WES can be used to inform walking policies and plans. The methodology of the study is shown in Fig. 5.

### Urban big data preprocessing

In this study, various urban data provided by Korea and Seoul were used. For the walkability index of previous studies, limited traffic infrastructure data such as intersection density was used. The length and the number of sidewalks were considered. The slope was calculated using the elevation of the area where the network directly belonged to the active travel category. In addition to the geometric and geographic characteristics of the walking network, data on land use, buildings, and household members, which may affect walkability, were also collected. Also, to account for public transport, each public transport stop and network was used. Public bicycle service in Korea is operated by each city. Seoul’s public bicycle provides various data on operation and use. Pedestrian satisfaction data collected in Seoul was used to verify the WES results. WES is created in a three-step sequence. First, all data are aggregated primarily on a 100 m spatial grid basis. Second, each data is converted to a z-score to remove unit effects. Finally, each data is integrated into the index of each category (WAI and TA). The integrated index finally becomes WES, which is the purpose of this paper. Table 2 lists the data used to calculate the walkability index and transit accessibility in this study. An attempt was made to utilize safety zone information and pedestrian entrance information data. However, due to limitations in other data coverage and accuracy, an index was developed based on the data presented in Table 2. Seoul is a city that implements various pedestrian policies. Since April 2021, speed limit lowering policies such as the safety speed 5030 policy have been enforced in South Korea to reduce fatalities and increase traffic safety^68^. Seoul is a representative city that implemented the safe speed 5030 policy. That policy affects a downward adjustment of the maximum speed limit to 50 km/h on Seoul’s main roads and 30 km/h on other roads to ensure the safety of pedestrians^69^.

**Table 2:** Data description.

	Description	Type	Preprocessing	Resource
Sidewalk	Length, width	Float	Sidewalk length	www.nsdi.go.kr
Intersection	Name, type	Categorical	Intersection density	www.nsdi.go.kr
Elevation	Elevation	Float	Slope in the urban area	www.nsdi.go.kr
Land use	Land use	Categorical	Entropy	www.nsdi.go.kr
Household	Count, average etc.	Float	Household density	https://sgis.kostat.go.kr/
Building	Building information	Float	FAR (floor area ratio)	www.nsdi.go.kr
Bus station	Name, line	Categorical	Network centrality	https://data.seoul.go.kr/
Subway station	Name, line	Categorical	Network centrality	https://data.seoul.go.kr/
Bike station	Name, line	Categorical	Network centrality	https://data.seoul.go.kr/
Seoul survey	Walking satisfaction	Float	Pedestrian satisfaction	https://data.si.re.kr/
Road	Road network	Categorical	Average road speed	https://www.ktdb.go.kr/
Trip	Origin/destin trips	Float	Traffic assignment	https://www.ktdb.go.kr/

### Multicollinearity

**Figure 6:**
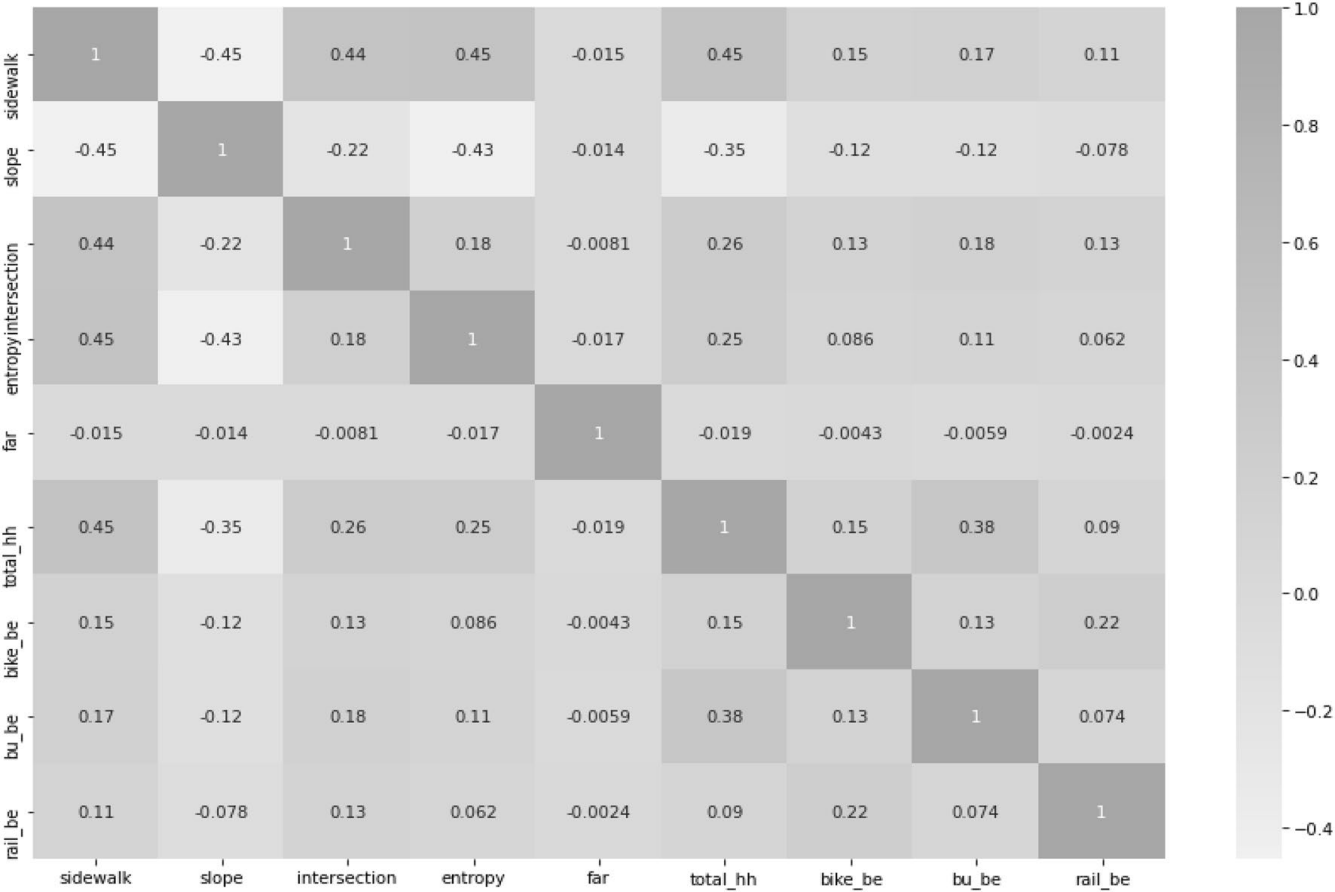
Correlation of variables.

Correlation refers to a linear relationship between two variables, and the closer it is to 0, the more there is no relationship between the two variables. Typical correlation values that have been suggested and used as the threshold range from 0.6 to 0.8^70^. The correlations between the variables used in this analysis are shown in Fig. 6.

WES should reflect factors that may affect walking. At the same time, it is necessary to review multicollinearity between variables because the overlapping of specific factors should not have a large impact on the evaluation. Variables with high multicollinearity were removed when constructing the index. If a variable with high multicollinearity is not preprocessed by integrating or removing it, the walkability value of the region containing the variable may appear higher than it is. The multicollinearity values of the variables are shown in Table 3.

**Table 3:** Variables VIF factor.

Variables	VIF factor
ENT (land use)	3.71163
Sidewalk	3.542793
Household	2.801861
Slope	1.59442
Intersection	1.514072
Bus centrality	1.445818
Bike centrality	1.180705
Rail centrality	1.114706

To identify multicollinearity in this study, the Variance Inflation Factor (VIF) was used to identify multicollinearity in the study. VIF is a criterion for judging whether an independent variable has a problem of multicollinearity. A VIF larger than 10 indicates multicollinearity. The VIF confirms that there is no multicollinearity problem in the given models as the values are in the range of an acceptable threshold value of 10^71^. A VIF between 5 and 10 indicates a high correlation that may be problematic^72^. All variables considered in this study were analyzed to have low multicollinearity, so we used all six major components when constructing the WAI.

### Walkability index (WAI) and transit accessibility (TA) model

This study developed an index for cities that want to walk through WAI and TA. This study differs from previous studies in that it systematized the evaluation through the classification of WAI and TA. The impact of each element of WAI and TA can be identified and reflected in the policy, and the relationship between walking and public transportation is further considered. The existing walkability is representative of Frank’s walkability method.

### Frank’s walkability (FWI)

Frank’s walkability (FWI) was calculated it as a weighted sum of three standardized (Z-scores) geographic factors: net residential density, intersection density and land use mix. Only the Z-score of intersection density was double-weighted^73,74^. The residential density is the ratio of the number of households in a neighborhood to the area of the neighborhood. The residential density can be calculated using demographic data. Intersection density is the number of intersections in the 1 km network buffer. This can be calculated by directly determining the intersection density within a region. For the land use mix, the entropy score was calculated using the area ratio of each of the three types of land use (residential, commercial, and office) to the total of the three types in the neighborhood. The land use index can be calculated in spatial grid units through land use information and building information.

### Walkability index components

The components of the walkability index in this study were sidewalk length, gradient, intersection density, entropy, FAR, and household density. Spatial aggregation was performed by extracting the length values included in the grid from the sidewalk data. The density of pedestrian infrastructure can be confirmed by the sum of the sidewalk lengths in each region. The elevation was based on Korea’s altitude data. The slope between adjacent points was calculated based on altitude data calculated in units of 90 m. When judging the minimum spatial interval of the provided data and the ease of interpreting the results, the 100 m grid unit was judged to be appropriate as the minimum spatial aggregation unit. Slopes can affect active travel, including walking. Steep slopes have a significant negative influence on walking attractiveness^75^. The higher the slope, the more negative the effect on walkability. For street design characteristics, the slope shows a negative association with walking volume^76^. Intersection density is the number of intersections in the grid. The number of intersections confirms the connectivity of a region. Entropy refers to the diversity of land uses. Land use and building data have one land-use classification value per polygon, and entropy is calculated using this value. If entropy is high, it can be expected that all normal activities can be performed within a small area^34^. The activity within a small area is closely related to walkability, as it is accompanied by walking. Entropy derived from the Shannon index was used^77^.

1 $$E=-\bigg(\sum_{i=1}^{n}{p}_{i}\times {\text{ln}}\left({p}_{i}\right)\bigg)/{\text{ln}}(n),$$

where $E$ is the entropy index, ${p}_{i}$ is the proportion of the neighborhood covered by the land use $i$ against the summed area for land use categories of interest, and $n$ is the number of land use categories of interest^77^. The floor area ratio (FAR) index is the ratio of commercial building area to total commercial land use area. A high index indicates that there is a significant proportion of small retail stores in the area. Places with a low FAR index tend to have large stores and shopping malls with large parking lots, so it is convenient to use a car for shopping. A high FAR index would be the opposite^34^. FAR calculations require commercial building and area information. The FAR index applied in this study is as follows. Household density refers to the density of households within a region. The index represents the density of households in urban areas. In this study, the walkability index is generated as the sum of the components z-score.

### The sum of z-scores of each component (WAI)

Each component was added to the z-score of the partial indices to create a walkability index. Because there is a unit difference for each variable, the z-score of each variable was calculated and added to create an index that removes the effect of the unit. For Frank’s walkability^32,73^, connectivity (intersection density) was given twice as much weight as other variables. The sidewalk and elevation, which comprehensively include the function of connectivity within the region, were considered in addition to intersection density, therefore, the walkability index was derived as the sum of z-scores without additional weights. In addition, this study confirms the walkability index evaluated in Seoul.

2 $$WAI= {Z}_{sl}+{Z}_{i}-{Z}_{s}+{Z}_{rs}+{Z}_{e}+{Z}_{h},$$

where $WAI$ is the walkability index, ${Z}_{sl}$ is the z-score of sidewalk length, ${Z}_{i}$ is the z-score of intersection density, ${Z}_{s}$ is the z-score of slope, ${Z}_{rs}$ is the z-score of road speed, ${Z}_{e}$ is the z-score of entropy, ${Z}_{h}$ is the z-score of household. In the case of the slope, the larger the value, the more negative the effect, so negative values were counted. As a result of this equation, the WAI was calculated.

### Transit accessibility (TA) components

Transit accessibility represents accessibility to public transportation within a region. Based on urban network analysis (or network centrality), the network centrality of stops included in the region is weighted and calculated spatially. Transit accessibility was derived from bus, subway, and bike stops and the network centrality of each stop. Betweenness centrality is an index that indicates the degree to which a node acts as a mediator or bridge along the shortest path. Therefore, a node with a high betweenness centrality (i.e., a station) will be an important point connecting routes in the network. The centrality within each network of bus, subway, and bike stations was calculated. Mobility hubs or transit stations can be installed in places with high betweenness value in Seoul to activate the mediating function of pedestrians and public transport.

### Urban network analysis and network centrality of each station

Betweenness centrality is the concept of a central node if it is between many different nodes, given that many shortest paths connecting nodes are passed. The betweenness centrality ($${{C}_{i}}^{B})$$ of node $$i$$ is expressed as Eq. (3).

3 $${{C}_{i}}^{B}=\frac{1}{(N-1)(N-2)}\sum_{j,k\in G,j\ne k\ne i}{n}_{jk}(i) / {n}_{jk}.$$

${n}_{jk}$ is the number of shortest paths between $j$ and k, and ${n}_{jk}(i)$ is the number of shortest paths between *j* and *k* including Node $i$*.* In this study, the number of bus, subway, and bicycle stops within a region was reflected by being weighted by the betweenness centralities. Because walking in a city is often carried out in connection with public transportation, transit accessibility is derived as the spatial sum of betweenness centrality weighted stations in the region. The weighted sum of stops can consider the network centrality as well as the number of stops. This will help derive a more appropriate TA because it can reflect the relative importance of stations in global transit networks. In addition, the installation of mobility hubs or transit stops at points with high bitterness values is associated with walkable cities with connected public transportation (e.g., implementing a 15-minute City). Betweenness centrality in railway networks is one of the measures that can change according to the introduction of new lines in the future, such as wide-area express railways. With the introduction of the wide-area express urban railway, many changes have been confirmed in the betweenness centrality value^78^. TA, which is one axis of the WES, can be evaluated, and the impact of pedestrian traffic on public transportation policies and development plans can be reflected.

### The sum of the z-scores of each component (TA)

The TA configuration was calculated as the sum of the z-score values of each configuration, as in the WAI. The z-score of each variable was calculated and added to create an index that removed the effect of the number of stops of each means and the unit of network centrality according to the network.

4 $$TA= {Z}_{bus}+{Z}_{bike}+{Z}_{rail},$$

where $TA$ is the TA index, ${Z}_{bus}$ is the z-score of the betweenness-weighted bus stations, ${Z}_{bike}$ is the z-score of betweenness-weighted bike stations, and ${Z}_{rail}$ is the z-score of the betweenness-weighted rail stations. In this study, it is assumed that each mode is equally important or preferred by pedestrians. In future studies, the mode split between transit and active transportation could be considered.

### Walkability evaluation system (WES)

Walkability Evaluation was performed by integrating the derived $WAI$ and $TA$. $WES$ directly reflects walking, and public transportation accessibility linked to walking is configured considering network centrality. The equation for calculating $WES$ is given as:

5 $${WES}_{i}= {WAI}_{i}+{TA}_{i},$$

where $WES$ is the WES, and ${WES}_{i}$ is the sum of *i*th $WAI$ and $TA$.

### Environmental benefits

Increasing the score of the WES can contribute to the activation of walking. There is a positive relationship between the magnitude of change in walkability and the changes in pedestrian volume and walking experience^79^. Also, walking is closely related to public transportation. To facilitate transit accessibility, walkways leading to rail stations should be given special attention in design with the aim of a pedestrian-friendly environment^75^. Transit users usually have more walking than non-users, and additional walking occurs when using public transportation^80^. Therefore, facilitating the use of public transport by improving the accessibility of public transport, i.e. increasing the accessibility of public transport, has a positive effect on the increase of pedestrian traffic, which in this study can help to increase the WES score as well as the comfort of walking public transport users. Therefore, public transportation activation policies directly increase walkability and macroscopically go along with the direction of decarbonization and net zero, which are global issues.

It is important to analyze the possibility of reducing carbon emissions from urban railway traffic^81^. This can be meaningfully applied to network reconstruction and expansion^82^. From the perspective of nodes and links, which will be constructed in the future, public transport activation policies for stations could bring additional environmental benefits from the energy-efficiency movement in cities as well as the transition to public transport. Policies that improve access to stations can contribute to the revitalization of public transportation. Accessibility and connectivity have significantly greater indirect effects on public transportation choices^83^. Using accessibility and connectivity, better mean prediction results were obtained when identifying captive users (people with essential public transportation)^84^. Scenario analysis was therefore carried out on the relationship between improving accessibility and key roles derived from the UNA. For most multimodal trips, the ratio (the ratio of travel time to total travel time) is in the modest range of 0.2–0.5^85^. Access time for the transit time was considered. The amount of method conversion was analyzed by considering the transfer distance and transfer distance when improving accessibility. The analysis was conducted by assuming a degree of improvement in accessibility (0.2–0.5), which is the ratio of the access time to the total travel time. It was confirmed that the traffic volume of passenger cars decreased and the traffic volume increased. Environmental benefits occur as vehicle traffic decreases. It presents the estimated environmental benefits of public TA improvements that can affect both walkability and access to public transport. Environmental benefits were calculated based on the amount of mode change due to improved accessibility to public transportation. The emissions factor table (Table 4) used to calculate the environmental benefits in this study is given as follows.

**Table 4:** Pollutant emission factors for private vehicles by speed (unit: g/km).

Speed	CO	NOx	VOC	PM2.5	CO2
10	1.51	0.59	0.15	0.02	380.85
20	0.8	0.38	0.07	0.01	251.48
30	0.57	0.3	0.04	0.01	197.3
40	0.45	0.25	0.03	0.01	166.1
50	0.38	0.22	0.03	0.01	145.34
60	0.33	0.2	0.02	0.01	130.33
70	0.29	0.18	0.01	0.01	118.85
80	0.27	0.17	0.01	0.01	109.73
90	0.25	0.15	0.01	0.01	102.27
100	0.23	0.15	0.01	0.01	96.03

*Resource* Appraisal Guidelines for Transport Facilities Investment.

This study also calculated the value of pollution cost savings (PS) through improvements in the TA, as shown in Eq. (6). The environmental benefit was calculated by applying the formula of Ku et al. for each link^48^. The emission factors according to the vehicle kilometers traveled (VKT) by mode and speed were used. Next, the unit cost of air pollution in Eqs. (7) and (8) was calculated by converting the extent to which each pollutant affects air pollution into costs according to modes and speed (Table 3). The benefit incurred by the mobility hub can be calculated daily and yearly during the implementation period.

6 $$PS={PS}_{after}-{PS}_{before},$$

7 $${PS}_{before}=\sum_{v=1}^{V}\sum_{l=1}^{L}{D}_{l}{E}_{l}^{v},$$

8 $${PS}_{after}=\sum_{v=1}^{V}\sum_{l=1}^{L}{D}_{l}{\prime}{E}_{l}^{v}.$$

${PS}_{before}$ and ${PS}_{after}$ are the values of pollution costs before and after TA improvements, respectively; ${D}_{l}$ and ${{D}{\prime}}_{l}$ are vehicle-km by link $l$, respectively, before or after transit accessibility improvements; $L$ is a finite set of links in Seoul; and ${E}_{l}^{v}$ is air pollution cost/km by link $l$ and by vehicle type by speed $v$. This study considered the mutually beneficial relationship between walking and public transportation. Improving walkability through public transportation contributes to the revitalization of public transportation. Analyzing the environmental benefits of improving public transport accessibility. This study improves accessibility for public transport located in areas with high WES scores. The environmental benefits were analyzed by applying pollutant coefficients.

## Results

### Walkability index (WAI) results in Seoul and Korea

The WAI results for Seoul and Korea were as follows. The components of the walkability index reflected in this study were sidewalk length, elevation, intersection density, entropy, floor area ratio (FAR), and household density. Therefore, areas with a high WAI (in red) can be said to be primarily walkable areas.

**Figure 7:**
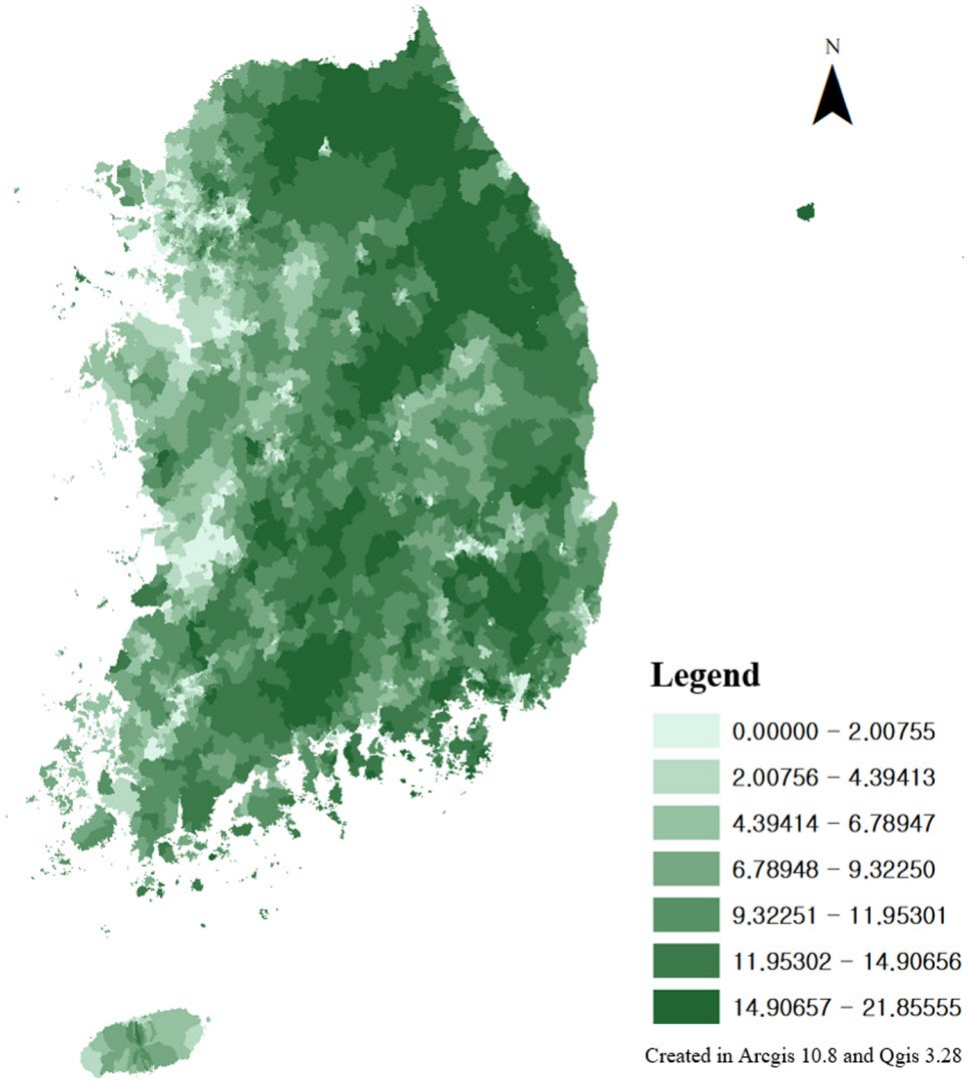
Gradient distribution in Korea, this map was generated in ArcGIS version 10.8 (https://www.esrikr.com/products/arcgis-desktop/) and Qgis desktop version 3.28 software (https://qgis.org/ko/site/).

**Figure 8:**
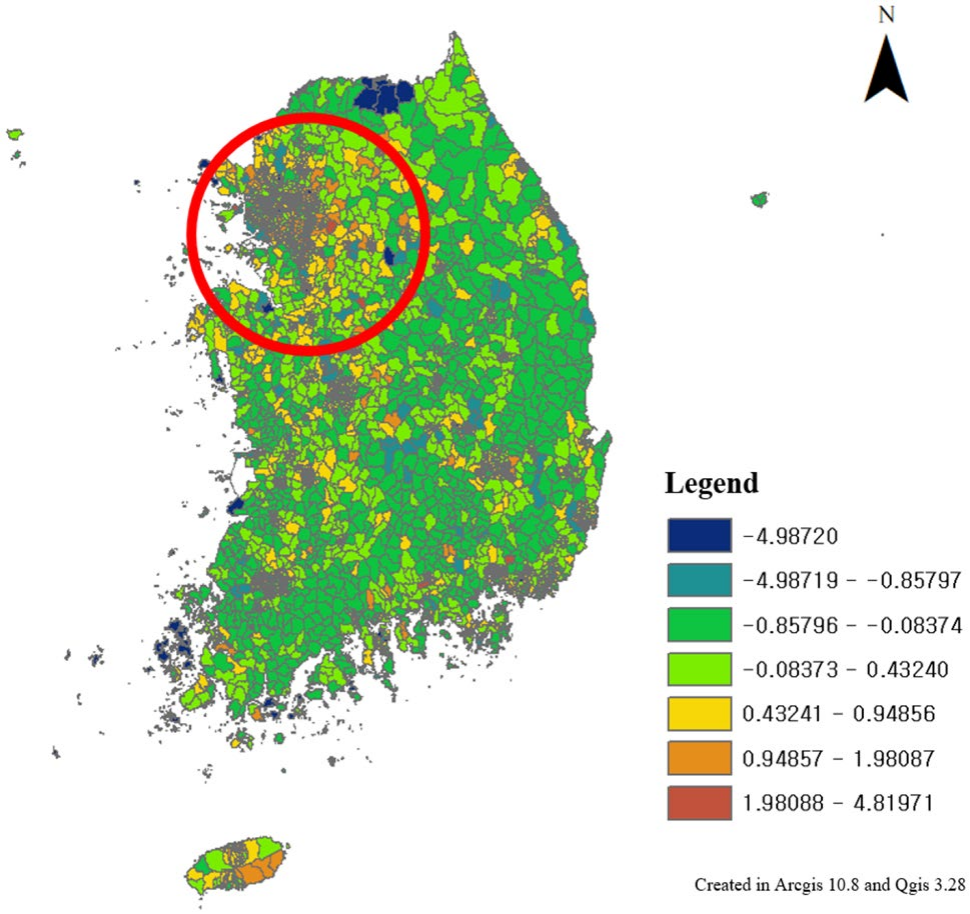
Average Household distribution in Korea, this map was generated in ArcGIS version 10.8 (https://www.esrikr.com/products/arcgis-desktop/) and Qgis desktop version 3.28 software (https://qgis.org/ko/site/).

**Figure 9:**
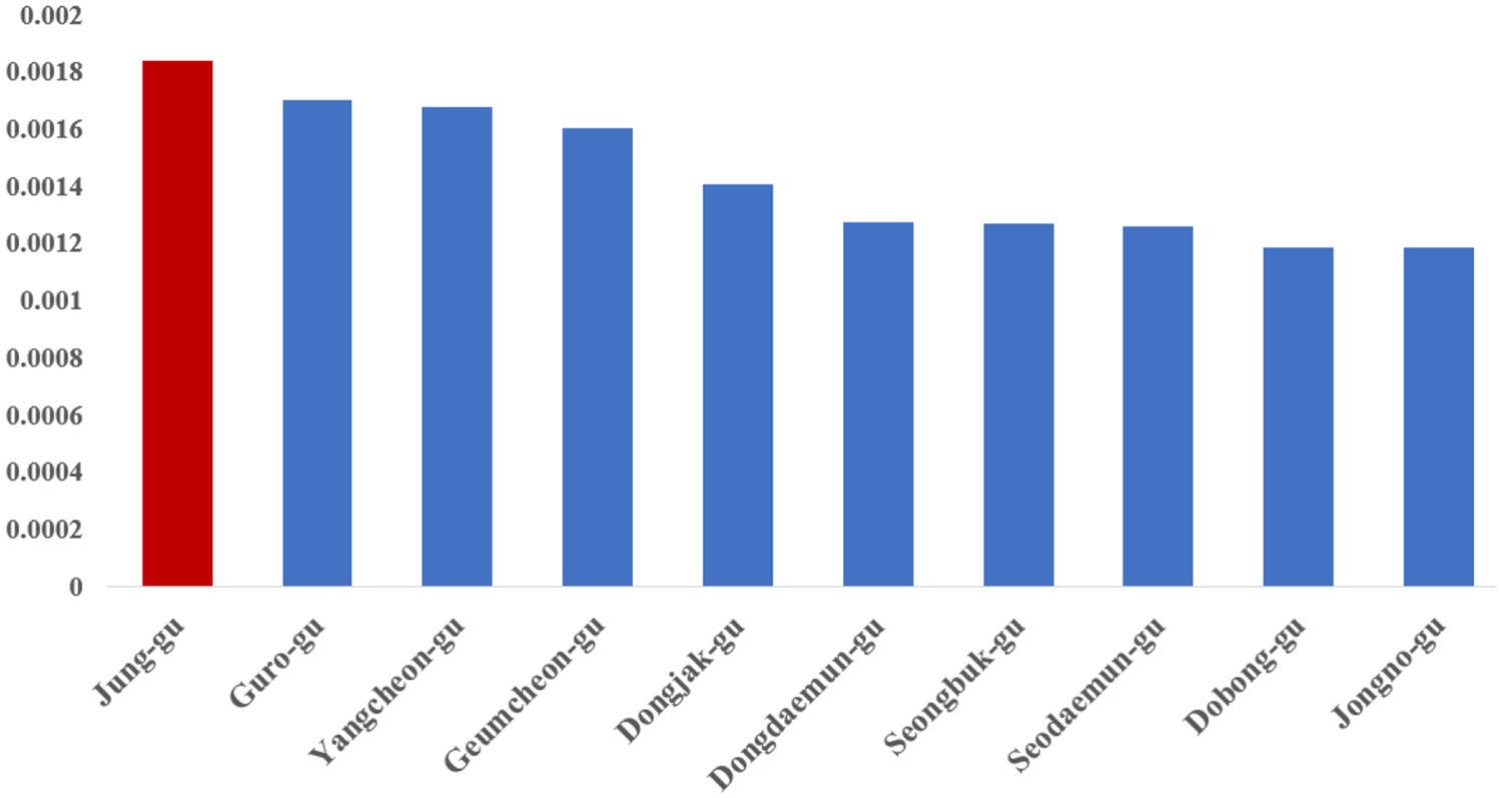
Top 10 Sidewalk density in Seoul, Korea.

**Figure 10:**
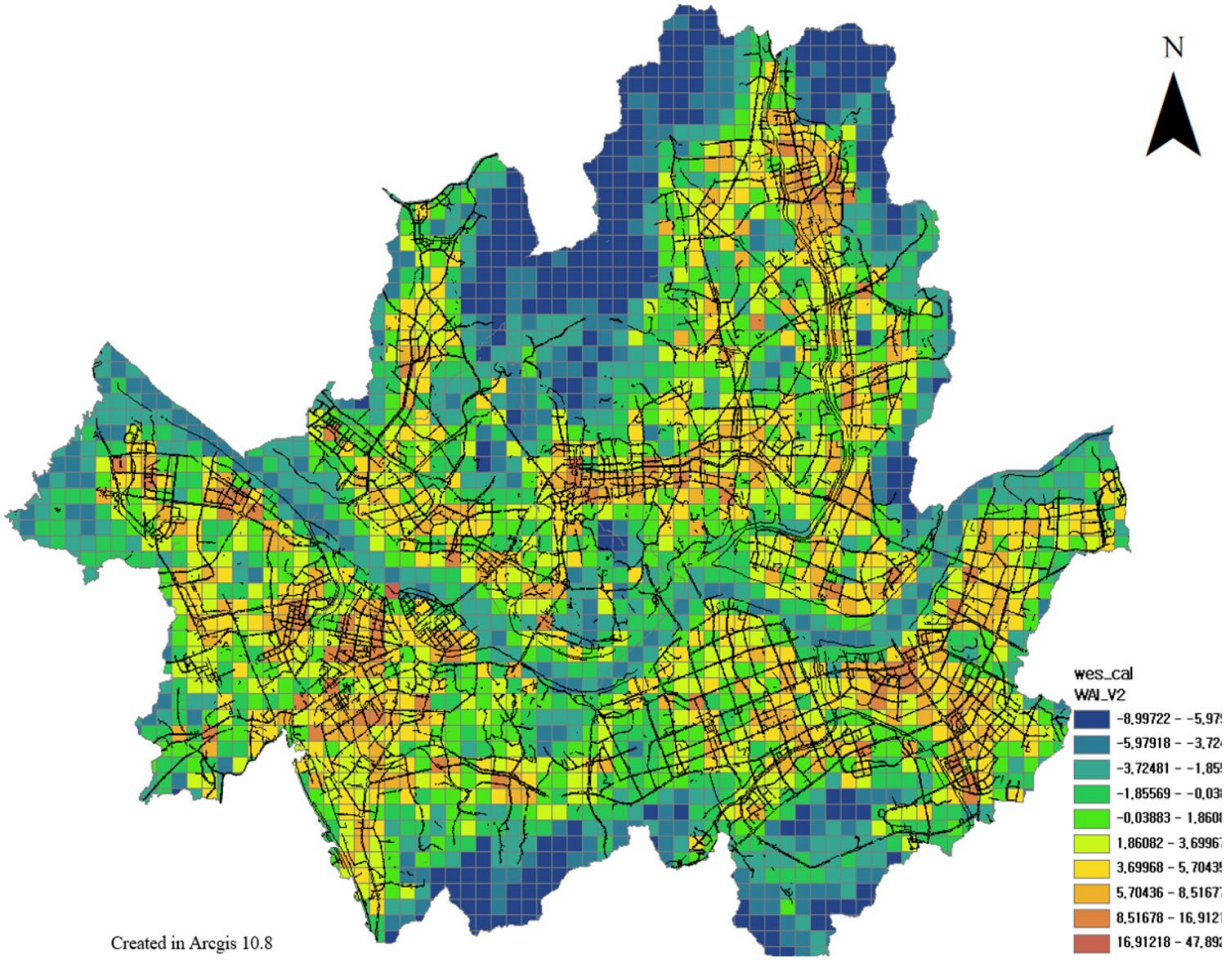
Walkability index (WAI) in Seoul, Korea, this map was generated in ArcGIS version 10.8 (https://www.esrikr.com/products/arcgis-desktop/).

The place with the highest WAI value in Korea is Seoul. It has many households and is a representative city that does not have a high slope as compared to Korea’s topography, which has a high slope due to its mountainous terrain (Figs. 7, 8). In addition, when looking at the region rather than the detailed grid, Seoul has a high value in Korea because it has a high value for all partial indices. This is because, in the case of mountainous regions, the components of WAI had the lowest overall score and a low slope score. Areas with high WAI scores included those with a large network of pedestrian paths, residential lots, or commercial facilities. The areas where the pedestrian network developed were those with the highest density of pedestrian paths in Seoul (Fig. 9). As a representative area with active green transportation policies in Seoul, projects on pedestrian roads are being carried out. It can be regarded as a representative area with a high WAI in Seoul by carrying out a large-scale pedestrian environment improvement project for a wide optical path (Fig. 10). In addition, it constitutes a green transportation promotion area and restricts the passage of vehicles that emit several pollutants; therefore, it is an area where citizens’ walkability can be high within the area. Areas with many households also have high pedestrian density. It can be confirmed that the WAI score is high according to the characteristics of each region, and the reliability of the WAI can be indirectly confirmed.

### Transit accessibility (TA) results in Seoul and Korea

The TA results for Seoul are as follows. The components of TA reflected in this study are bus station, rail station, and bike station betweenness. The network centrality values of buses and bikes are distributed relatively globally. The value train centrality is concentrated in the center of Seoul. In the case of train links, these differences may occur because they are limited compared with general roads. The TA of Seoul was calculated by integrating the betweenness of the three means as follows.

**Figure 11:**
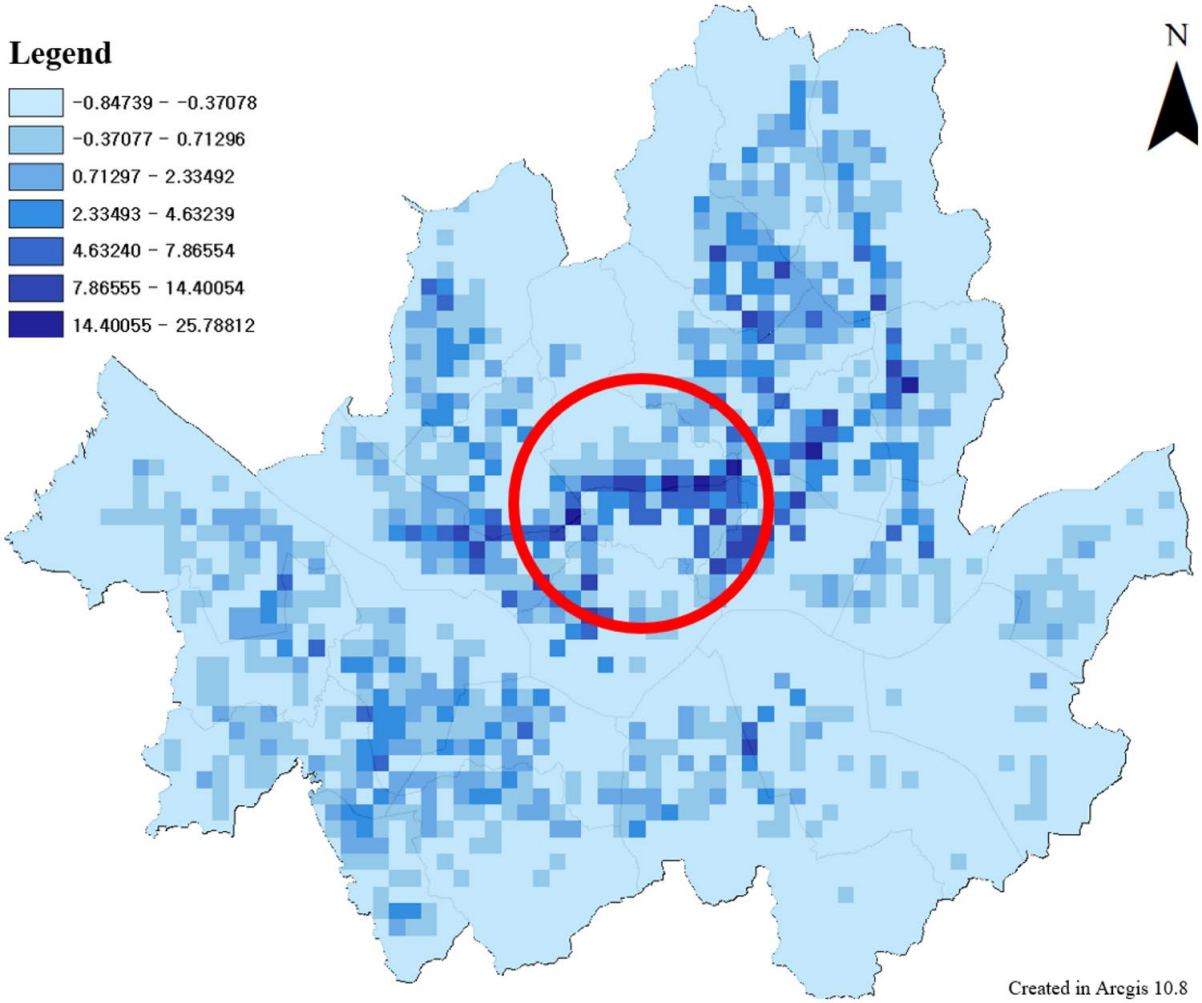
Transit Accessibility in Seoul, this map was generated in ArcGIS version 10.8 (https://www.esrikr.com/products/arcgis-desktop/).

**Figure 12:**
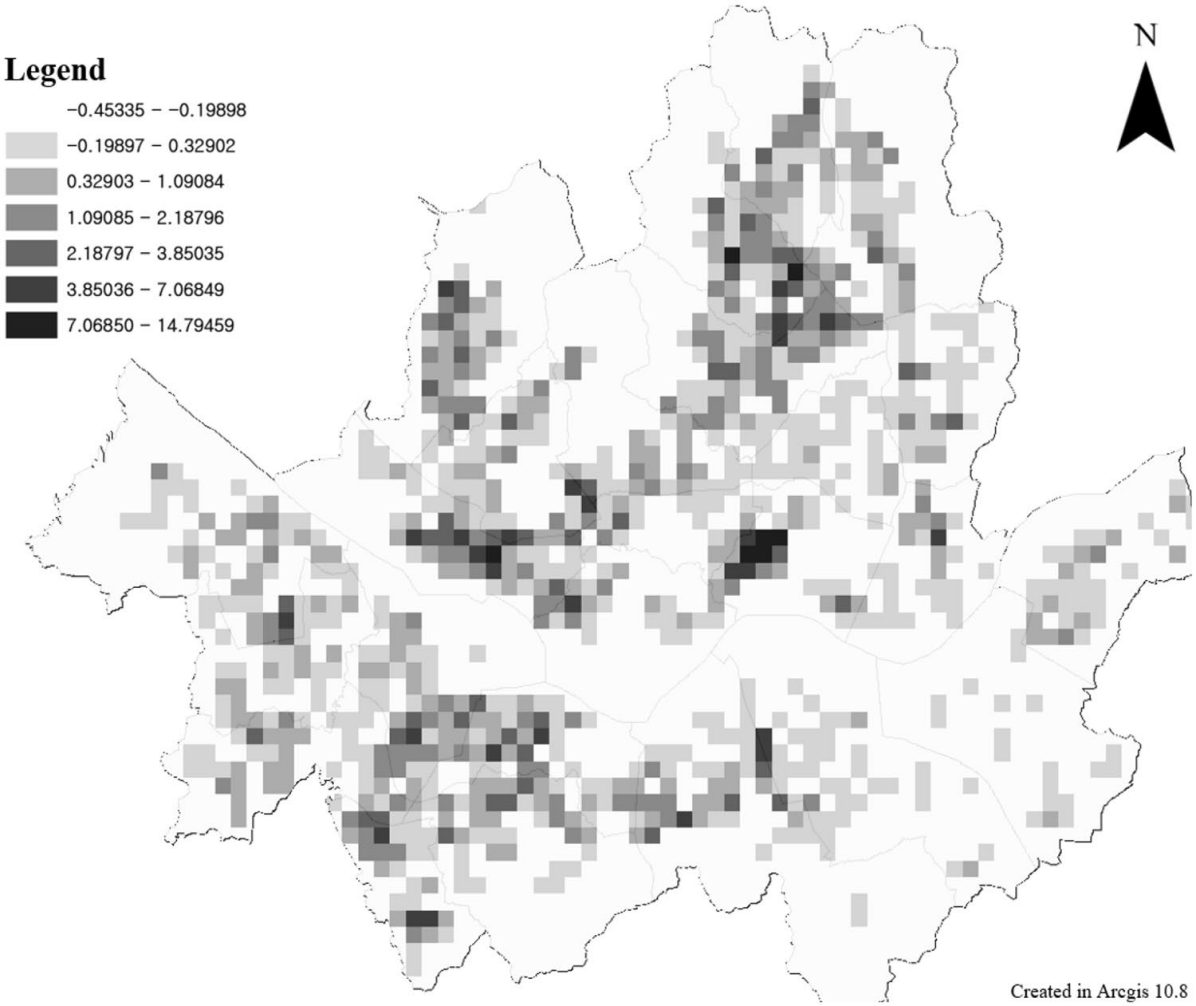
Bus stations (betweenness weighted z-score), this map was generated in ArcGIS version 10.8 (https://www.esrikr.com/products/arcgis-desktop/).

**Figure 13:**
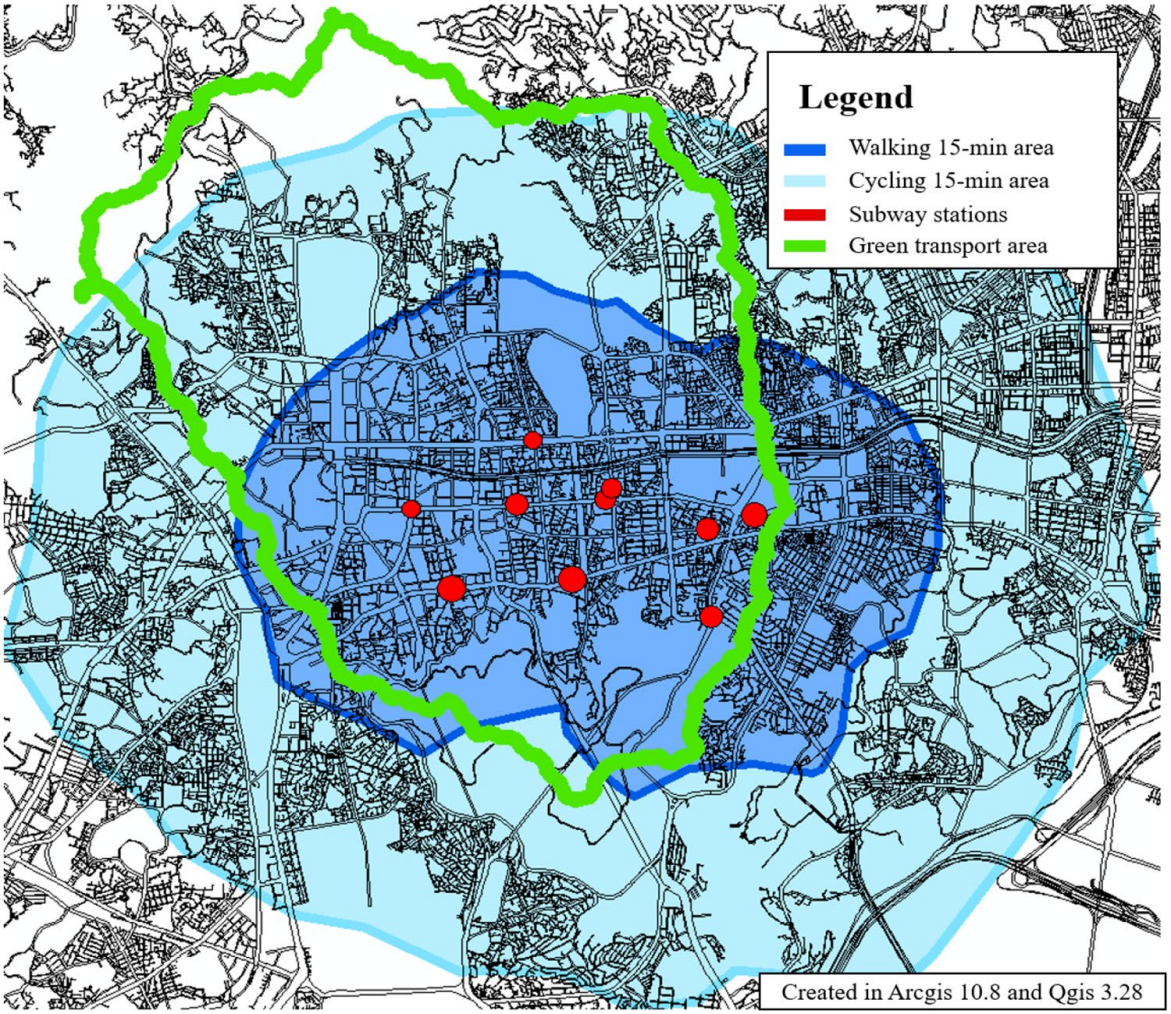
Advanced 15-minute City area, this map was generated in Qgis desktop version 3.28 software (https://qgis.org/ko/site/).

**Figure 14:**
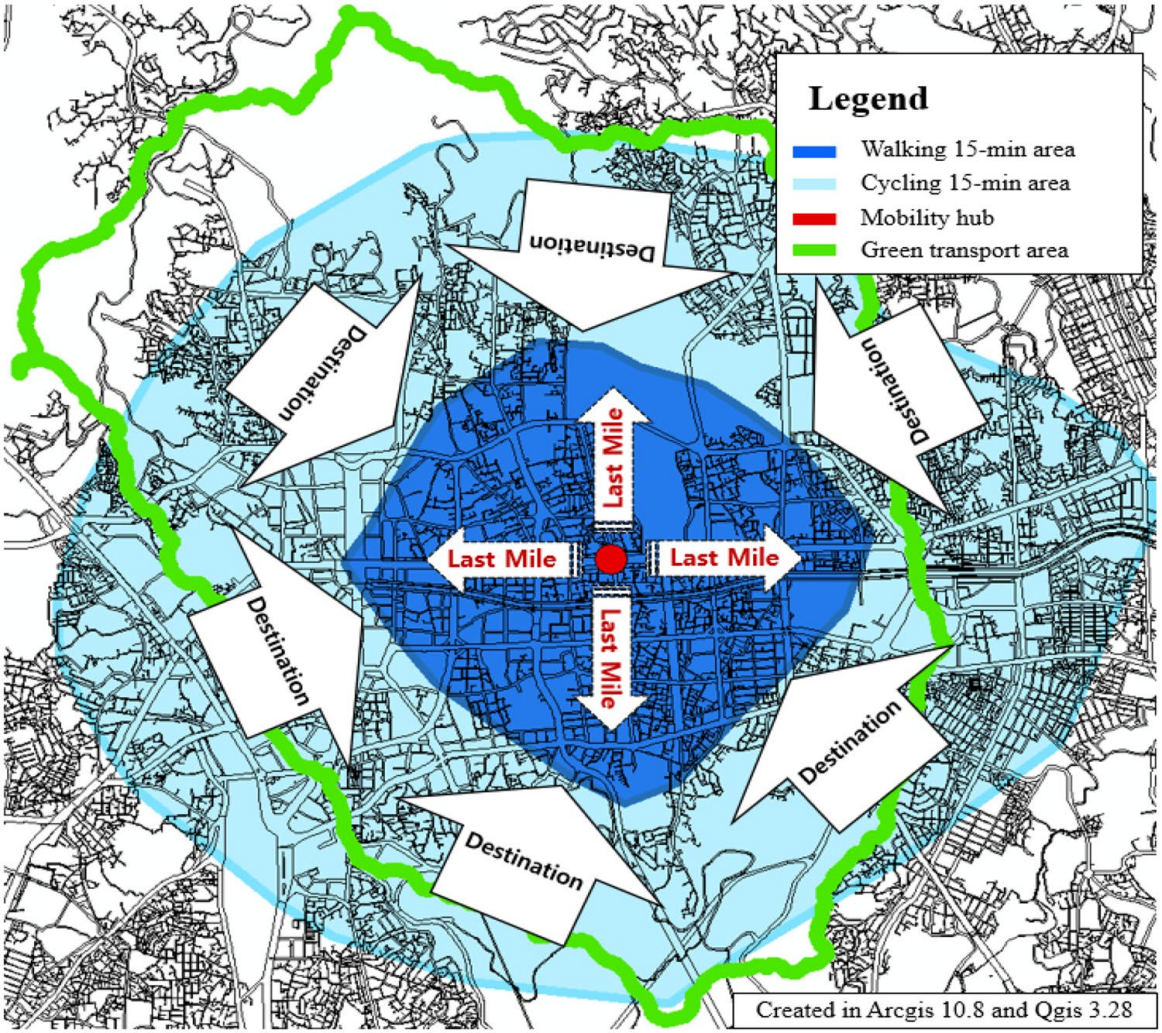
Mobility hub example, 15-minute City area, this map was generated in Qgis desktop version 3.28 software (https://qgis.org/ko/site/).

The overall distribution of the TA values in Seoul (Fig. 11) was like the centrality distribution of the bus stations (Fig. 12). In the case of the central part, it appears that the high-value distribution of TA has changed because of the consideration of trains and bicycles. The results of TA in Seoul were analyzed to show that the Betweenness value of the old town is high. This area includes Seoul’s green transportation area and is also an area where many transfer stations exist in Seoul. It is also a historical district in Seoul, and it is an area that can induce citizens to pass through. TA value improvement for these areas can increase the ease of travel for leisure purposes as well as commuting. Station improvements or transit stations, such as mobility hubs^86^, can be built around high-value areas. The area that can be walked or biked to within 15 min of a metro station within the green zone is shown in Fig. 13. If a mobility hub is installed within the green area, the accessible area is shown in Fig. 14. After moving to the Mobility Hub, citizens can easily move through the congested city center on foot and by bicycle.

Betweenness centrality is a measure of the role of intermediaries between origin (O) and destination (D). For a pedestrian-oriented metropolis, connectivity with public transportation is essential. The degree of intermediation of a public transport station can be measured by its betweenness value. Where the value is high, stations such as mobility hubs should be created.

### Walkability evaluation system (WES) results in Seoul and Korea

**Figure 15:**
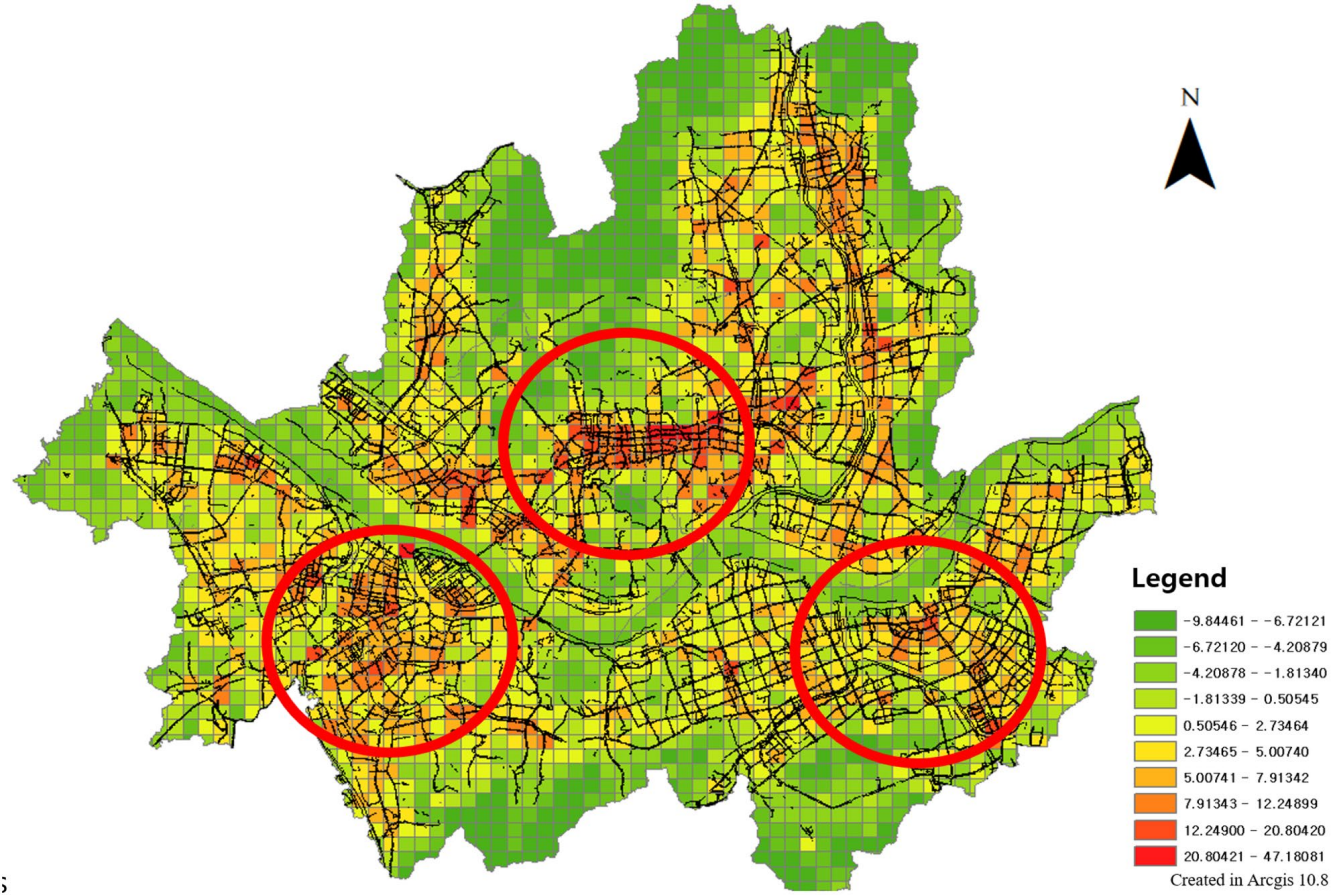
Walkability Evaluation System in Seoul, this map was generated in ArcGIS version 10.8 (https://www.esrikr.com/products/arcgis-desktop/).

The WES is a model that integrates a walkability index and transit accessibility. Each index is built and integrated to consider the elements of walking and public transportation. A configured system can be applied, either globally or locally. The evaluation system of this study can evaluate unbalanced walkability, even within the same regional unit, by evaluating the walkability of specific regions. In the case of walking traffic, because the travel distance is short, it was judged that the evaluation of small regional units was more significant for walking traffic, so it was composed of detailed regions. Furthermore, it is configured in small regional units such that it can be directly combined with the walking amount or walking environment score for each link. Seoul’s walkability was evaluated based on an established evaluation model (Fig. 15).

Areas, where the high value of WAI coincided with the high value of TA, tended to show relatively more prominent WES (Table 5). This result is more appropriate, considering the significant impact of public transportation on walking. In Table 5, The central area of Seoul, where green traffic promotion areas and pedestrian projects are active, had high WES scores, and when the average WES score was checked by area, it was found to be the highest area (Jung-gu). The average WES scores of the two districts (Yeongdeungpo-gu and Songpa-gu), which are representative business and commercial centers of Seoul, were also highly evaluated. In the case of central Seoul, there are many public transportation transfer facilities, and the high degree of development of factors such as walking infrastructure and slope resulted in a high WES score.

**Table 5:** Top 10 regions in average WES score in Seoul.

ID	Name	Total WES score	Grid	Avg WES score
11,140	Jung-gu	237.6587	36	6.60163
11,230	Dongdaemun-gu	214.93	54	3.980186
11,560	Yeongdeungpo-gu	314.9047	98	3.213313
11,200	Seongdong-gu	199.4178	67	2.976385
11,470	Yangcheon-gu	208.2064	74	2.8136
11,440	Mapo-gu	254.2049	100	2.542049
11,710	Songpa-gu	262.3466	146	1.796895
11,215	Gwangjin-gu	84.2691	73	1.154371
11,530	Guro-gu	92.20947	98	0.940913
11,260	Jungnang-gu	77.41018	85	0.910708

### WES validation: Moran’s I, GWR

**Figure 16:**
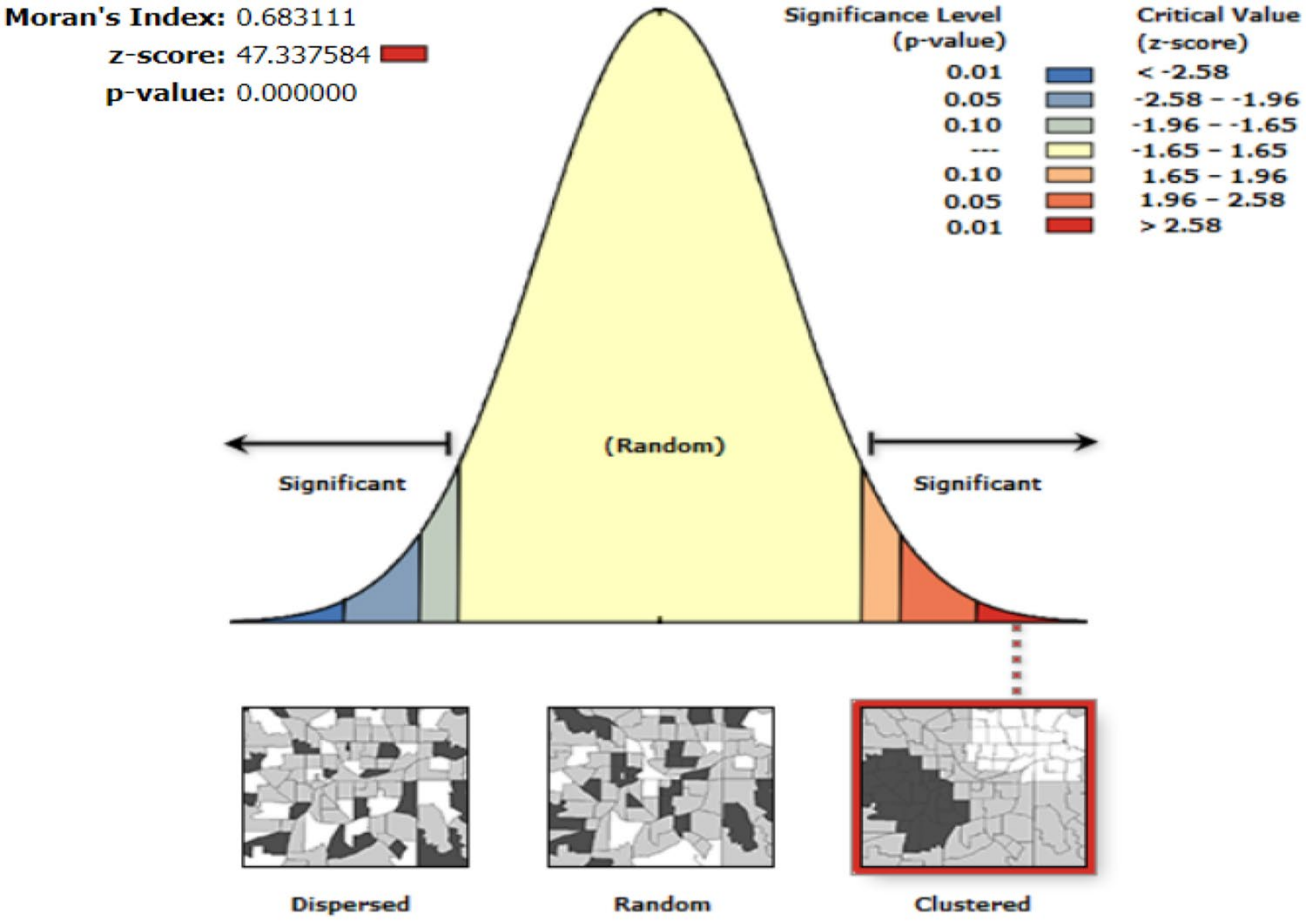
Moran’s I Index results.

Because the WES values were collected locally, they were regionally clustered. In previous studies, the walkability index was verified through comparison with citizens’ survey data or the value of the walk score. In this study, the explanatory power of the WES value for walking satisfaction in Seoul was confirmed. First, the spatial autocorrelation of the walking satisfaction data for Seoul was confirmed. The autocorrelation of gait satisfaction variables was confirmed using Moran’s I method. The pedestrian satisfaction data appeared as Moran’s I value (= 0.68), which can be judged to be clustered, and the p-value was significant (Fig. 16).

**Figure 17:**
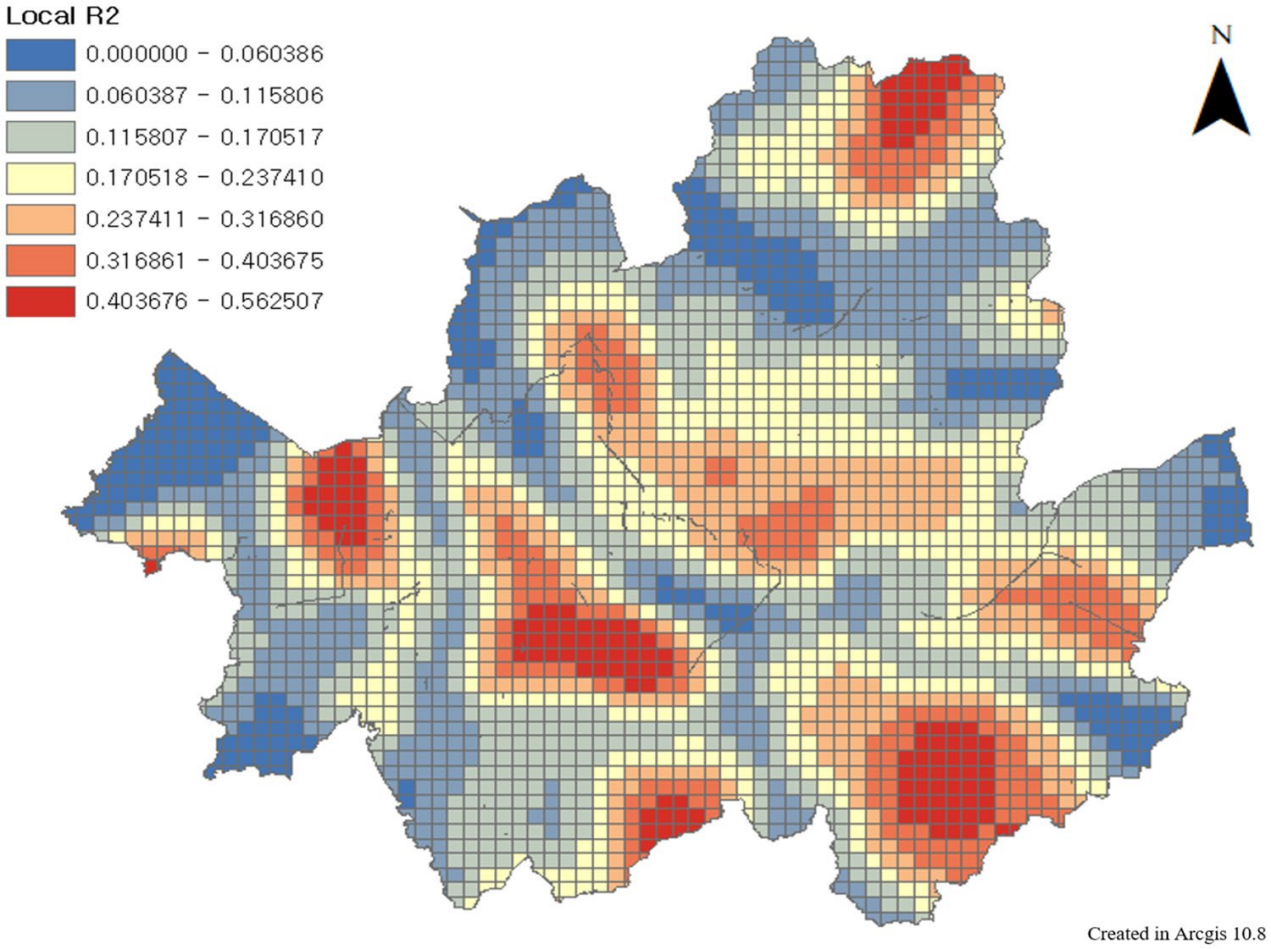
Geographically weighted Regression results, this map was generated in ArcGIS version 10.8 (https://www.esrikr.com/products/arcgis-desktop/).

Therefore, a geographically weighted regression method was applied to confirm the explanatory power of the spatially correlated variables with the locally collected explanatory variables. When constructing the index, the values of all components were integrated into a z-core; thus, when checking the explanatory power, a geographically weighted regression analysis was performed with the values of the original variables (Fig. 17). Geographically weighted regression is an analysis that confirms the degree of influence of each explanatory variable on geographically correlated dependent variables. It is possible to check the degree of influence of each regional variable as well as the explanatory power of the entire model.

The results of the geographically weighted regression analysis showed that the global explanatory power (${R}^{2}$) was 0.75 and the p-value was significant. Therefore, WES has explanatory power for pedestrian satisfaction, which means that the evaluation system can explain gait satisfaction for each element. The explanatory power (${R}^{2}$) of each region is diversely distributed up to 0.56, which is seen as a limitation of the spatial range of the pedestrian satisfaction data used to verify the WES model in this study.

### Environmental and health benefits

#### Case study 1: safety speed 5030 policy effect analysis

**Figure 18:**
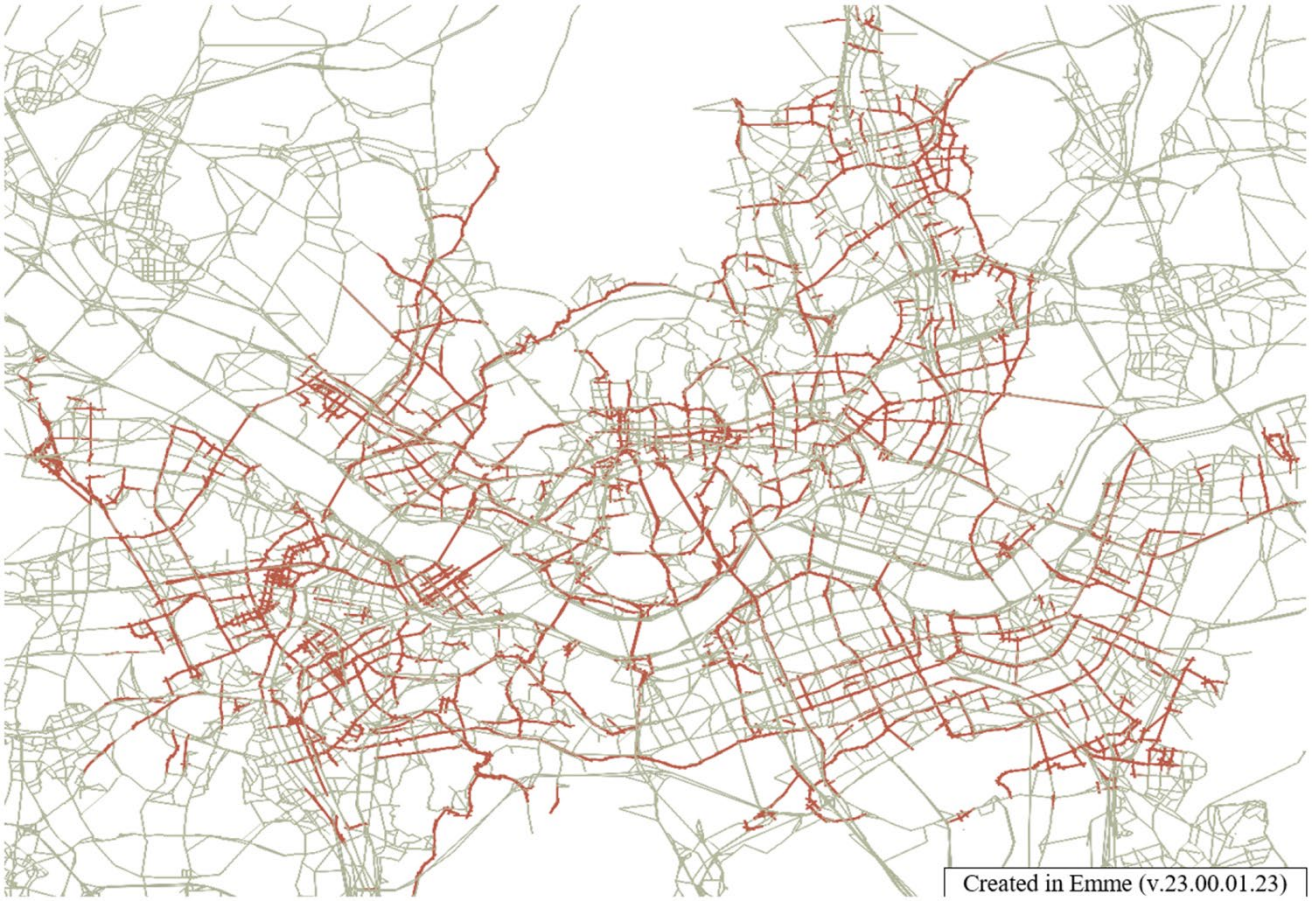
Edit Road networks in EMME, this map was generated in EMME desktop version 23.00.01.23 (https://education.bentley.com/).

**Figure 19:**
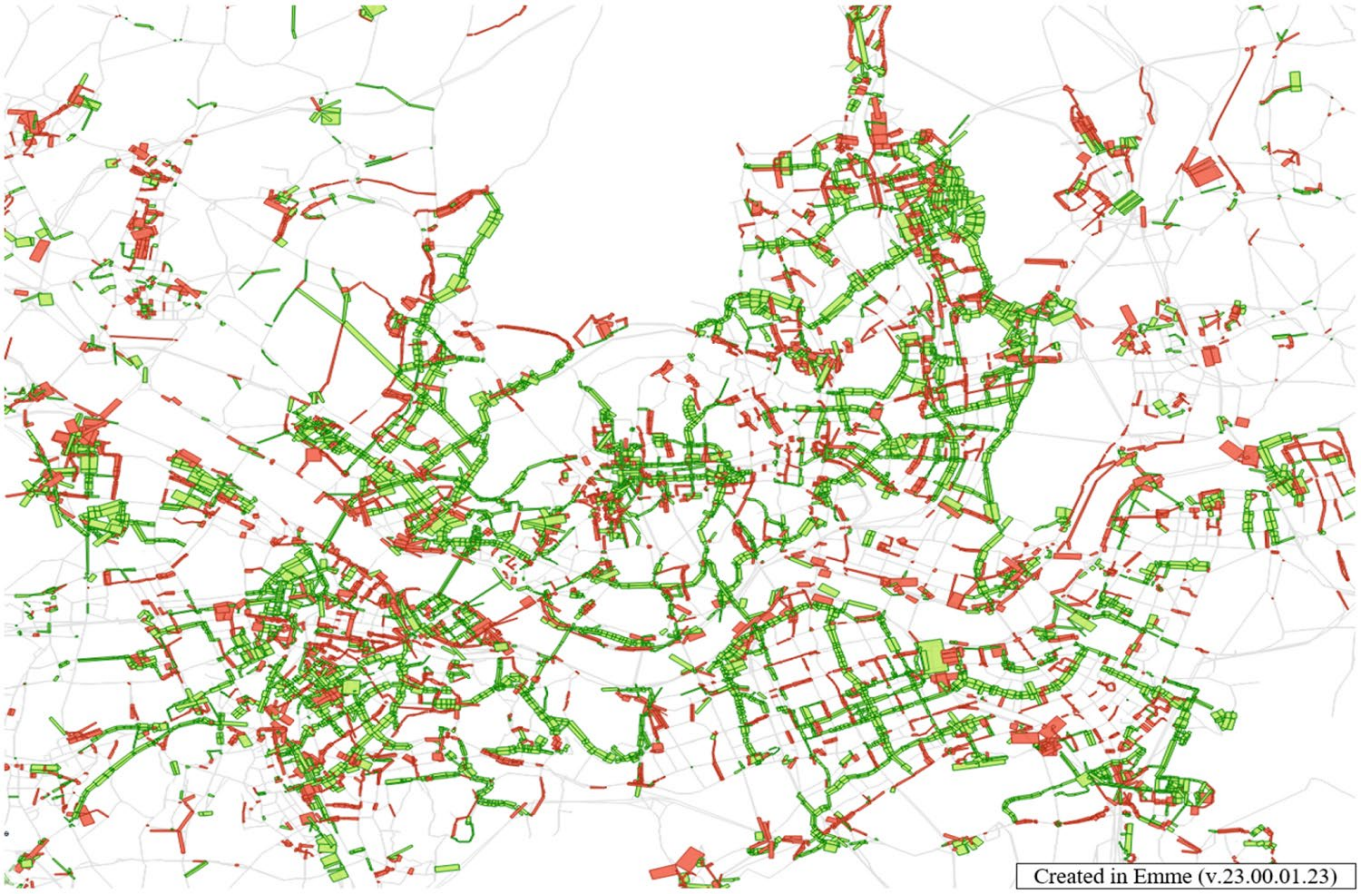
Reduced Road traffic in Seoul, this map was generated in EMME desktop version 23.00.01.23 (https://education.bentley.com/).

The Safe Speed 5030 law reduces the speed limits for all city roads to 50 km/h and 30 km/h for back roads. The speed of vehicles negatively impacts walkability. Roads with speeds exceeding 50 km/h are deemed unsuitable for pedestrians^87^. Applying the safety speed 5030 to Seoul roads (Fig. 18) can yield the results depicted in Fig. 19. A reduction in traffic has been observed in the city center. This can potentially enhance the pedestrian walkability within the city center.

On the other hand, pollutant emissions increased. The environmental side-benefits of applying the policy to the network can be analyzed. The speed, traffic volume, and emission factor for each speed were calculated for each link. Road speed restrictions have increased pollutant emissions. Pollutant emissions results from the Safe Speed 5030 policy are shown in Table 6. The Safe Speed 5030 policy showed significant pollutant increases.

**Table 6:** Environmental costs in Seoul by safety speed 5030 (unit: t).

	CO	NOx	VOC	PM2.5	CO_2_
Before	49,163.20	13,615.66	13,242.17	185.97	7,860,147.68
After	51,376.81	14,024.67	14,275.26	185.10	8,086,052.69
Difference	2213.60	409.01	1033.09	− 0.87	225,905.01

Therefore, the safety speed 5030 policy is a policy with two sides. Reduced speed and traffic in the city center contributed to the improvement of walkability. However, it has a negative impact on environmental benefits. It is essential to consider these aspects when making policy decisions for a city.

#### Case study 2: public transportation accessibility improvement scenario

The access time for the transit time was considered. The amount of method conversion was analyzed by considering the transfer distance and transfer distance when improving accessibility. It was confirmed that the traffic volume of passenger cars decreased, and the traffic volume increased. As vehicle traffic decreases, environmental benefits occur. The amount of pollutant reduction is calculated based on these criteria. In this study, the environmental benefits of improving accessibility to public transportation, particularly in areas with high WES, were analyzed. The results are shown in Table 7. Due to improved accessibility to public transportation stations, CO_2_ emissions decreased by about 344,887.23 tons in Seoul. There are environmental benefits to the city as a result of improved walkability and increased accessibility.

**Table 7:** Environmental benefits in Seoul by improvements of transit accessibility (unit: t).

	CO	NOx	VOC	PM2.5	CO_2_
Before	49,163.20	13,615.66	13,242.17	185.97	7,860,147.68
After	48,237.80	13,496.28	12,638.85	187.90	7,515,260.45
Difference	− 925.40	− 119.38	− 603.32	1.93	− 344,887.23

## Conclusions

This study proposes a comprehensive walkability evaluation system that can be comprehensively applied by measuring and integrating the walkability index and transit accessibility. Pedestrian-oriented policies, visions, and goals require quantitative evaluation. A walkability assessment method that can be comprehensively applied to pedestrian-oriented cities (e.g., Paris, a 15-minute City) is proposed. Focusing on betweenness, public transportation in the old town, the last mile area were checked. The walk accessibility area was reviewed after the installation of a public transport station or mobility hub in a high-value location. Actions on climate change and carbon neutrality are being urged all over the world. As transportation is a major factor in carbon emissions, it is urgent to solve it. To respond positively in the transportation field, active travel and public transport are attracting attention. The importance of public transportation is recognized by many planners^88^. The need for public transportation, including the environment^52,53^ and safe walking, is presented. The study has developed a system to assess the walkability of policymakers and citizens. A more straightforward index was developed by employing various data from cities that have been recently produced in WES.

This study verifies the multicollinearity (VIF) of the variables to be examined for the index. Walkability Index considers pedestrian characteristics, intersection density, slope, entropy, far, and household. The created index can be used to evaluate the walkability index (WAI) score of each region. Furthermore, this study examines the network centrality of bus stops through various methods. TA is calculated by utilizing network centrality. Betweenness is an index to be considered as it has a mediating function in a city. TA considering betweenness is necessary for the evaluation of large cities or pedestrian cities considering public transport. WES score is evaluated for each grid. You can check the distribution of the WES value. The WES score can also indirectly check the topographical distribution of Seoul. Finally, this study is meaningful in establishing a Walkability Evaluation System (WES) model composed of WAI and TA and evaluating walkability in urban areas. The WES model is verified through Seoul City’s pedestrian satisfaction data. The global explanatory power of the WES model for walking satisfaction in Seoul was significant with an R-square of 0.75. Therefore, the WES model is shown to work in evaluating walkability in urban areas. In addition, the environmental benefits of walkability improvement scenarios were identified. It is possible to quantitatively confirm how much convenience pedestrian convenience can provide through the calculation of benefits from increased accessibility to public transportation.

This study analyzed the effect of policies for walking. It was analyzed that the safe speed 5030 policy reduced downtown traffic. The policy was also analyzed to have pedestrian and bicycle environmental impacts. Improving public transportation accessibility can contribute to the vitalization of walkability and public transportation. When analyzing the effects, environmental benefits can also be identified. Policymakers can make cities more walkable by curbing car demand or boosting public transport. This is positive for walkability, environmental factors, and personal health. Therefore, it can be judged that the city’s plans and policies centered on walkability are policies that conform to Net Zero. The elements selected in this paper are representative data that are likely to be provided by most cities. Therefore, the index of this paper can be applied worldwide. However, the spatial aggregation unit of data may vary depending on the scope provided by the city. In addition, the evaluation should be applied according to the characteristics of the city. For example, in the case of a city with a small area and a small number of people, a Paris model centered on walking and cycling can be applied. Walkability assessment can be centered on WAI. For a city with a large area or a city with developed public transportation, it is appropriate to evaluate WAI and TA in considerations.

Assessing pedestrian mobility, including public transport access, can serve as a foundation for pedestrian policies and initiatives. To achieve carbon neutrality, policy grounds to be applied in the transportation sector can be presented. Furthermore, with increasing social demand and policies for pedestrian-friendly traffic management, road space reorganization, promoting walkability, and securing pedestrian spaces, this study’s evaluation framework is expected to have multifaceted applications. The WES of this paper can promote the use of transportation in response to climate change. It can help establish transportation policies and suggest new directions for the city. To respond to global boiling, it will be a WES that can be applied to cities such as Paris, Barcelona, Portland, and Seoul. However, this paper has the following limitations. First, directly considering the width of the pedestrian path or the number of pedestrians passing through the pedestrian path can be an additional criterion for the pedestrian infrastructure to be improved. Second, when integrating indicators, weights can be added by applying the methodology of multi-criteria analysis to factors that are considered more important. Third, the index will be further developed if measures that can realistically consider the capacity or status of public transportation are added.

## Data Availability

The datasets used and/or analyzed during the current study are available from the corresponding author on reasonable request.
